# Species Identification and Orthologous Allergen Prediction and Expression in the Genus *Aspergillus*

**DOI:** 10.3390/jof11020098

**Published:** 2025-01-27

**Authors:** Maria C. Zuleta, Oscar M. Gómez, Elizabeth Misas, Susana Torres, Álvaro L. Rúa-Giraldo, Juan G. McEwen, Ana M. Garcia, Clayton L. Borges, Orville Hernández, Angela M. López

**Affiliations:** 1 Cellular and Molecular Biology Unit, Corporación para Investigaciones Biológicas (CIB), Medellín 050034, Colombia; mariac.zuleta@udea.edu.co (M.C.Z.); oscarm.gomez@udea.edu.co (O.M.G.); elizabeth.misas@gmail.com (E.M.); susana.torres@udea.edu.co (S.T.); alvaro.rua@udea.edu.co (Á.L.R.-G.); juan.mcewen@udea.edu.co (J.G.M.); ana.garciac@udea.edu.co (A.M.G.); orville.hernandez@udea.edu.co (O.H.); 2 School of Medicine, Universidad de Antioquia (UdeA), Medellín 050010, Colombia; 3 School of Microbiology, Universidad de Antioquia (UdeA), Medellín 050010, Colombia; 4 Environmental Microbiology, Research Group, School of Microbiology, Universidad de Antioquia (UdeA), Medellín 050010, Colombia; 5 Faculty of Pharmaceutical and Food Sciences, Universidad de Antioquia (UdeA), Medellín 050010, Colombia; 6 Genomic and Proteomic Center, Laboratório de Biologia Molecular, Universidade Federal do Goiás, Goiânia 74690-900, Brazil; clbluiz2@gmail.com; 7 MICROBA Research Group, School of Microbiology, Universidad de Antioquia (UdeA), Medellín 050010, Colombia

**Keywords:** *Aspergillus*, genomic identification, orthologs, allergens

## Abstract

The genus *Aspergillus* comprises a diverse group of fungi that can cause a range of health issues, including systemic infections and allergic reactions. In this regard, *A. fumigatus* has been recognized as the most prevalent allergen-producing species. This genus taxonomic classification has been subject to frequent updates, which has generated considerable difficulties for its classification when traditional identification methodologies are employed. To demonstrate the feasibility of this approach, we sequenced the whole genomes of 81 *Aspergillus* isolates and evaluated a WGS-based pipeline for precise species identification. This pipeline employed two methodologies: (i) BLASTn web using four barcode genes and (ii) species tree inference by OrthoFinder. Furthermore, we conducted a prediction of allergenic capacity based on a homology analysis across all the isolated species and confirmed by RT-qPCR the expression of three orthologous allergens (Asp f 1, Asp f 3 and Asp f 22) in fifteen different *Aspergillus* species. The species-level identification rate with the barcoding and the species tree were calculated at 64.2% and 100%, respectively. The results demonstrated that *A. fumigatus*, *A. flavus* and *A. niger* were the most prevalent species. The species *A. hortae*, *A. uvarum*, *A. spinulosporus*, *A. sydowii*, *A. westerdijkiae*, *A. amoenus* and *A. rhizopodus* identified in this study represent the inaugural report of their presence in our region. The results of the homology analysis indicated the presence of orthologous allergens in a wide range of non-*fumigatus* species. This study presents a novel approach based on WGS that enables the classification of new species within the genus *Aspergillus* and reports the genomic sequences of a great diversity of species isolated in our geographic area that had never been reported before. Additionally, this approach enables the prediction of allergens in species other than *A. fumigatus* and demonstrates their genetic expression, thereby contributing to the understanding of the allergenic potential of different species within this fungal genus.

## 1. Introduction

*Aspergillus* is a fungal genus with a multifaceted nature and a cosmopolitan distribution across ecosystems and climatic zones [1]. The genus has significant industrial and food relevance [2,3,4,5] as well as the potential to cause health issues in humans and animals. The clinical manifestations of the fungus range from localized to severe infectious processes with frequently lethal invasive forms (invasive pulmonary aspergillosis). Furthermore, it plays a significant role as an environmental allergen, causing a variety of allergic respiratory diseases including allergic rhinitis, sinusitis, severe asthma with fungal sensitization (SAFS) and allergic bronchopulmonary aspergillosis (ABPA) [6,7,8].

Approximately 446 species have been described within the *Aspergillus* genus, classified in six subgenera, 27 sections and 75 series [9]. Among the species considered pathogenic, *A. fumigatus* (*Fumigati* section) is the most ubiquitous in the environment, the most virulent and the main one associated with disease in humans and animals. It is followed by *A. flavus* (*Flavi* section), *A. niger* (*Nigri* section), *A. terreus* (*Terrei* section) and *A. nidulans* (*Nidulantes* section), with new species emerging [10]. 

The molecular identification of this fungal genus has been primarily carried out by *in silico* multi-locus sequence typing (MLST) [11] and the sequencing of the genome regions of nuc rDNA internal transcribed spacer rDNA region (ITS1- 5.8S-ITS2) for identification at the genus/section level and of Calmodulin (cmdA), β-tubulin (benA) and RNA polymerase II (rpb2) for identification at the species level [9,12,13]. In recent years, an alternative to the rapid sequencing a few markers is whole genome sequencing (WGS), which has been successfully implemented to identify species [14]. Furthermore, it provides all the information required for pathogen typing and characterization at the species level, as well as the identification of genes of interest (e.g., antimicrobial resistance genes and virulence factors as allergenic genes) in a shorter time (3–5 days) [15]. 

To date, 38 allergens are officially recognized in the *Aspergillus* species by the WHO/IUIS Allergen Nomenclature Sub-Committee [16]. A total of 30 allergens have been described in *A. fumigatus* (Asp f 1–f 13, Asp f 15–f 18, Asp f 22, Asp f 23, Asp f 27–f 29 and Asp f 34–f 39). Due to the structural homology between Asp f 15 and Asp f 13, as well as Asp f 16 and Asp f 9, a proposal has been made to reduce the number to a total of 28 allergens [17,18]. Considering other *Aspergillus* species, three allergens in *A. niger* (Asp n 14, Asp n 18 and Asp n 25), two in *A. oryzae* (Asp o 13 and Asp o 21) and a single allergen in *A. flavus* (Asp fl 13), *A. versicolor* (Asp v 13) and *A. terreus* (Asp t 36) have been described. Some of these allergens are known for their structural, toxic and enzymatic roles, and their relationship to virulence has been reported [19,20,21]. 

Comparative genomics using BLASTp has revealed the presence of orthologous allergens in various fungal genera [22] and specifically for the genus *Aspergillus*, it is known that *A. nidulans* and *A. oryzae* present homologous genes to the allergens recognized in *A. fumigatus* [23]. This indicates that other species may possess proteins with the potential to act as allergens or to cross react. However, this remains an unknown field for most of the species within this fungal genus. Thus, the identification of *Aspergillus* species with allergenic capacity becomes increasingly important, due to the rise in the diversity of recently described or reclassified species according to the current taxonomy for this genus and the limited number of sequences described in recently classified and potentially allergen-producing species.

In this work, we designed a whole genome sequencing-based pipeline for determining a precise species identification through the barcode analysis of four genes, ITS, cmdA, benA and rpb2, and the phylogenomic reconstruction inferred by OrthoFinder using *ab initio* protein prediction, as a proof of concept. Additionally, an allergenic capacity prediction of *Aspergillus* species was conducted based on homology. Finally, the expression of the Asp f 1, Asp f 3 and Asp f 22 genes was evaluated, employing RT-PCR as described for the expression of the *A. fumigatus* allergens [24].

## 2. Materials and Methods

### 2.1. Aspergillus Isolates

The *Aspergillus* strains were obtained from the MicroCIB 223 strain collection (Corporación para Investigaciones Biológicas Medellín—Colombia). The isolates were previously collected from different intra- and extra-hospital environments and from clinical samples from individuals with aspergillosis. Ethics committee approval was obtained from the local Ethics committee of the Corporación para Investigaciones Biológicas. A total of 81 isolates were utilized in this study. They were previously identified to the section level, according to their microscopic and macroscopic characteristics in peptone-dextrose agar (PDA), malt extract agar (MEA), Sabouraud dextrose agar (SB) and Czapek yeast autolysate (CYA) agar culture media, by the taxonomic keys available for the genus [25,26,27]. The description of the isolates employed in this study is presented in Table S1.

### 2.2. Nucleic Acid Extraction

For whole genome sequencing, genomic DNA (gDNA) was extracted from 81 isolates cultured in brain heart infusion (BHI) broth at 30 °C and shaken at 120 rpm. The biomass was collected during the exponential growth phase after 96 h of incubation. Cell lysis was performed through mechanical disruption with liquid nitrogen and pestle and mortar. DNA was obtained using phenol/chloroform extraction method [28] and RNA was eliminated by treatment with RNase A (Thermo Fisher Scientific, Inc., Waltham, MA, USA) for 3 h at 37 °C. Furthermore, the integrity was assessed by 1% agarose gel electrophoresis.

To evaluate allergen gene expression, RNA was extracted from 15 isolates of different species (Table S2) cultured in PDA at 27 °C for 7 days. Subsequently, the colonies were collected, and the mechanical disruption of the mycelium was performed using liquid nitrogen. The total RNA was isolated using TRIzol (Thermo Fisher Scientific, Inc., Waltham, MA, USA) and the E.Z.N.A. TOTAL RNA KIT I (Omega Bio-tek, Inc.) according to manufacturer’s instructions. Contamination with genomic DNA was removed from the RNA samples by a treatment with DNase I (Thermo Fisher Scientific, Inc., Waltham, MA, USA) for 120 min at 37 °C. To confirm the absence of contamination from genomic DNA, a conventional PCR was carried out amplifying the β-tubulin gene [29]. The quality and concentration of nucleic acids were evaluated using a NanoDrop 2000 spectrophotometer (Thermo Fisher Scientific, Inc., Waltham, MA, USA).

### 2.3. Genome Sequencing

Library preparation and 150-bp paired-end sequencing were performed using next-generation sequencing (NGS) on the Illumina HiSeq Xten PE 150 platform (BGI Inc., Hong Kong, China). The samples were sequenced on two lanes of the Illumina platform, resulting in approximately seven million pair-end reads per isolate and an average genome coverage of 30×. The raw data are available on the Sequence Read Archive (SRA) website from NCBI (BioProject PRJNA975750).

### 2.4. Pre-Assembly Analysis

The quality of the Illumina reads was assessed through analysis using the FastQC v0.11.9 tool with the default settings [30]. Sequences exhibiting low quality and adapter contamination were subjected to trimming using Trimmomatic v 0.39 [31].

### 2.5. De Novo Assembly

The high-quality reads (Q score > 30) were assembled *de novo* using the SPAdes v3.1.1 pipeline with the BayesHammer module for error correction, iterative k-mer lengths (21, 33, 55 and 77 bp) and the “careful” option [32]. Scaffolds with a length of less than 500 base pairs were excluded from further analysis. The quality of the draft genome assembly was evaluated using QUAST v.5.0.2 [33], comparing the metrics with the representative genomes. Subsequently, the quality of each genome was verified by following the next three parameters: average coverage of paired-end reads, histograms of the distribution of the percentage of Guanine–Cytosine (GC%) and length of sequence alignments.

### 2.6. Molecular Genomic Identification

The molecular identification of *Aspergillus* isolates was performed by extracting ITS, cmdA, benA and rpb2 sequences in the assemblies using the local BLASTn algorithm. Subsequently, the FASTA sequences were uploaded to the BLASTn web search tool for comparison with the database sequences Nucleotide Collection (nr/nt) of NCBI and EMBL-bank. The most significant matches were selected based on a threshold of >95% identity and a minimum read length coverage of >80%.

### 2.7. Phylogenetic Tree

Isolates that showed a 100% identity percentage with two or more species underwent phylogenetic analyses by section. A combined dataset of cmdA, benA and rpb2 gene sequences from the studied isolates and reference species was used (accession numbers can be found in Table S3). Sequence datasets were aligned using MAFFT v7.505 [34] for each gene and then combined into a concatenated matrix by section. Finally, phylogenetic trees were inferred using the Maximum Likelihood (ML) method with the IQtree v2.0.3 program [35], applying an Ultrafast Bootstrap adjustment of 1000 replicates. *A. ochraceus* and *A. robustus* were the outgroup species in both phylogenies. Phylogeny visualization and editing were performed using FigTree v1.4.4 and Inkscape v1.2.1.

### 2.8. Genome Annotation

The resulting assemblies were annotated by *ab initio* prediction with the Augustus v3.4.0 program [36] using *A. oryzae*, *A. fumigatus*, *A. nidulans* and *A. terreus* as the precomputed gene model.

### 2.9. Gene Homology and Species Tree Analysis

The predicted proteins from genome annotation were subjected to an analysis by sequence homology with the OrthoFinder v2.0.9 pipeline [37] (with default settings) for comparison of the protein sets of the *Aspergillus* representative genomes reported in the databases (Table S4) and annotated proteomes from this study. 

The genomic identification was carried out using the species tree output obtained from gene homology analysis. OrthoFinder identifies orthogroups, which are then used to infer gene trees and, subsequently, to construct a rooted species tree [37]. The visualization and editing of the tree were carried out in FigTree v1.4.4 and Inkscape v1.2.1. A total of 29 allergens were filtered from the orthogroup output. The sequences were extracted from annotated proteomes and the identity percentage was assigned to each allergen ortholog. The genomics workflow is illustrated in Figure S1.

### 2.10. Allergen Expression Analysis

The expression of the Asp f 1, Asp f 3 and Asp f 22 genes was evaluated using Reverse Transcription PCR (RT-PCR). For this, total RNA, extracted and purified, was employed for the synthesis of cDNA using Reverse Transcription KIT (SMOBIO Technology, Inc., Neuenburg am Rhein, Germany) in accordance with the manufacturer’s instructions.

The primers were designed from the sequences obtained in this study during the genomic analyses of the conserved regions using Geneious Prime software version 2022.2.2. Universal primers were designed for each section when sequencing homology allowed; if this was not the case, specific oligonucleotides were designed for each species (Table S5). The thermodynamic parameters of the primers, Guanine-Cytosine content, melting temperature (Tm) and potential to form secondary structures were analyzed and studied *in silico* using OligoAnalyzer Tool (Integrated DNA Technologies, Inc., Coralville, IA, USA, https://www.idtdna.com/pages/tools/oligoanalyzer, accessed on 20 October 2022) [38]. The genomic DNA of *Aspergillus* spp. was employed as a template used to standardize the PCR conditions and evaluate primer specificity.

To evaluate the expression of the genes under investigation, a gel-based RT-PCR was conducted using the thermal cycler SimpliAmp (Thermo Fisher Scientific, Inc., Waltham, MA, USA) and Hot-start polymerase (Thermo Fisher Scientific, Inc., Waltham, MA, USA) according to the manufacturer’s instructions. The thermocycling conditions included an initial denaturation for 10 min at 95 °C, followed by 40 cycles of 15 s for denaturation at 95 °C, 30 s for annealing at 57 °C and 30 s for extension at 72 °C. Three technical replicates were conducted for each reaction. Negative and positive controls (DNA of each species evaluated) were employed for each of the PCR runs. As internal control, the endogenous reference gene 18S ribosomal RNA (18 rRNA) (endogenous standard) was evaluated for each sample. Amplification products were subjected to electrophoresis on a 2.5% agarose gel at 70 volts for 150 min.

## 3. Results

### 3.1. Genome Assembly, Analysis and Identification of Aspergillus Isolates

The complete genome sequences of 81 *Aspergillus* isolates that were previously identified at the section level based on morphological characteristics are described in this study. The isolates were classified into seven sections *(Fumigati*, *Flavi*, *Nigri*, *Terrei*, *Circumdati*, *Nidulantes* and *Clavati*) (Table S1). The initial classification was employed to verify the genome size and GC content.

First, the quality of the genome assemblies was compared based on genome size, GC content and the number of assembled contigs (Table S6, column b). In general, the genome sizes were observed to range between 28 Mb and 39 Mb and the GC percentages between 47.1% and 52%, corresponding to the section to which the isolates belonged. The MCA-4 isolate was classified within the *Fumigati* section and exhibited a GC% of 49% (similar to the other *A. fumigatus* isolates). However, its genome size was 6 Mb larger than expected, but being within the possible range for the genus *Aspergillus*, it was included in the genomic identification and subsequent orthological analysis.

The statistics obtained from the Augustus and OrthoFinder analyses were included (Table S6, column c). The *ab initio* predictions showed a range of 11,586 to 15,613 predicted protein-coding genes, with a high proportion (>95.4%) of homologs found in the genus. The genomes with more than 500 contigs in their assembly (isolates marked with asterisks in Table S6) exhibited a 14.8% (n = 12) rate, which could potentially impact the quality of the assemblies in terms of continuity and integrity. However, it was observed that 11 of the 12 genomes contained more than 95% of the benchmarking single-copy universal orthologs (Table S6, column c), and only one of them, with 81% corresponding to MCA-4, was not included in the further analysis. Therefore, the genome annotation of these 11 isolates was of high enough quality for gene content comparisons.

### 3.2. Barcode Analysis for Species Identification

**Figure 1:**
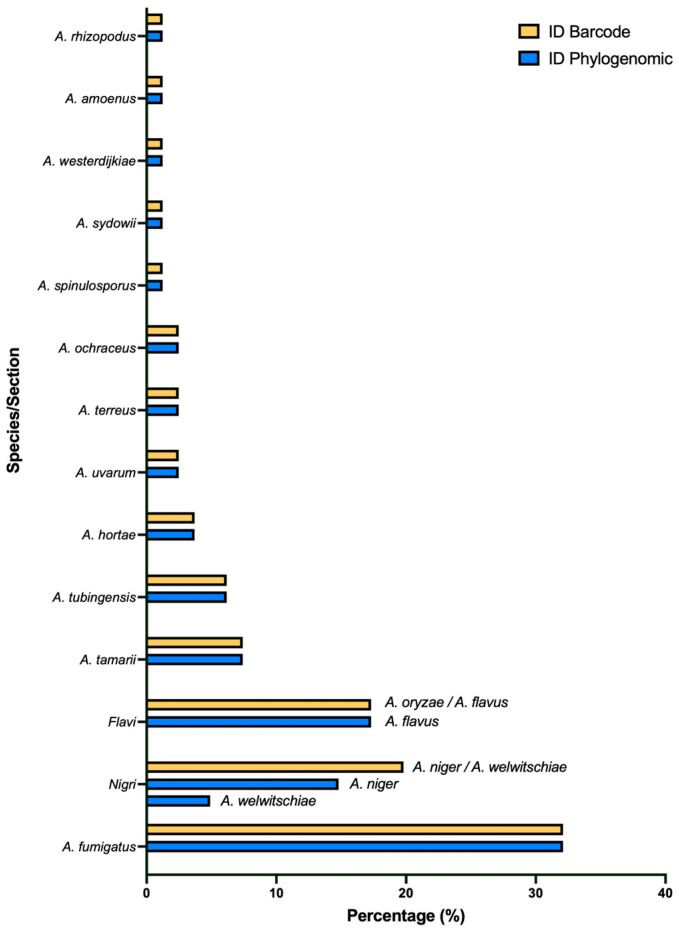
Identification of *Aspergillus* isolates. Yellow, molecular identification by barcode analysis; blue, phylogenomic identification by reconstruction based on predicted proteomes. Species on the right are the indistinct ones with barcodes which were resolved with phylogenetic identification.

The sequence of the markers used for fungal DNA barcoding analysis in *Aspergillus* [9] (ITS, cmdA, benA, rpb2) was initially identified within the assemblies using local BLASTn. A single copy of each gene was found in each genome assembly. A BLASTn search (https://blast.ncbi.nlm.nih.gov/Blast.cgi, accessed on 30 June 2021) comparing these sequences with the ones in the databases was conducted to determine the species. The platform enabled the attainment of a resolution at the species level in 64.2% (n = 51) of the isolates (Figure 1). However, 17.3% of the isolates presented ambiguity between the species *A. flavus* and *A. oryzae*. The remaining isolates (19.8%) exhibited ambiguous results between the species *A. niger*, *A foetidos*, *A. awamorii*, *A. ficuum*, *A. welwitschiae*, *A. phoenicis* and *A. brasiliensis*. However, only *A. niger*, *A. welwitschiae* and *A. brasiliensis* are currently accepted as species of the *Nigri* section [9]. Furthermore, *A foetidos*, *A. ficuum* and *A. phoenicis* correspond to synonyms of the species *A. niger* [39,40,41,42], while *A. awamorii* is a synonym of *A. welwitschiae* [35]; thus, the non-differentiation in this classification was reduced only to the species *A. niger*, *A. welwitschiae* and *A. brasiliensis.*

The application of the BLASTn web to these four markers did not appear to be sufficient for the taxonomic resolution of the species to which these isolates belong; therefore, this group was treated as the *Nigri* section and the *Flavi* section. It is important to note that not all isolates from these two sections shared the same issue regarding classification. The species *A. tamarii* (belonging to the *Flavi* section), *A. tubingensis* and *A. uvarum* (belonging to the *Nigri* section) were successfully resolved at the species level.

Due to the lack of species-level resolution in 36% of the isolates, the phylogenetic analyses by section were conducted to attempt a taxonomic classification at the species level. Two phylogenetic inferences were performed based on the concatenated sequences of the cdmA, benA and rpb2 regions to determine the relationships among the isolates classified as belonging to the *Flavi* and *Nigri* sections. The molecular phylogenies obtained are illustrated in Figures S2 and S3.

In both phylogenies, an insufficient phylogenetic signal was observed to differentiate the isolates with ambiguous classifications as distinct species. The isolates previously classified as *Aspergillus* section *Flavi* were grouped within the monophyletic cluster of *A. flavus/A. oryzae*. Of the isolates identified as *Aspergillus* section *Nigri*, 12 were grouped with *A. niger* species and 4 were grouped with *A. welwitschiae*. However, it is important to note that these two taxa also resolve into a monophyletic clade, and once again, the combined use of these four markers was insufficient to assign the isolates to a specific species.

### 3.3. Phylogenomic Species Identification

**Figure 2:**
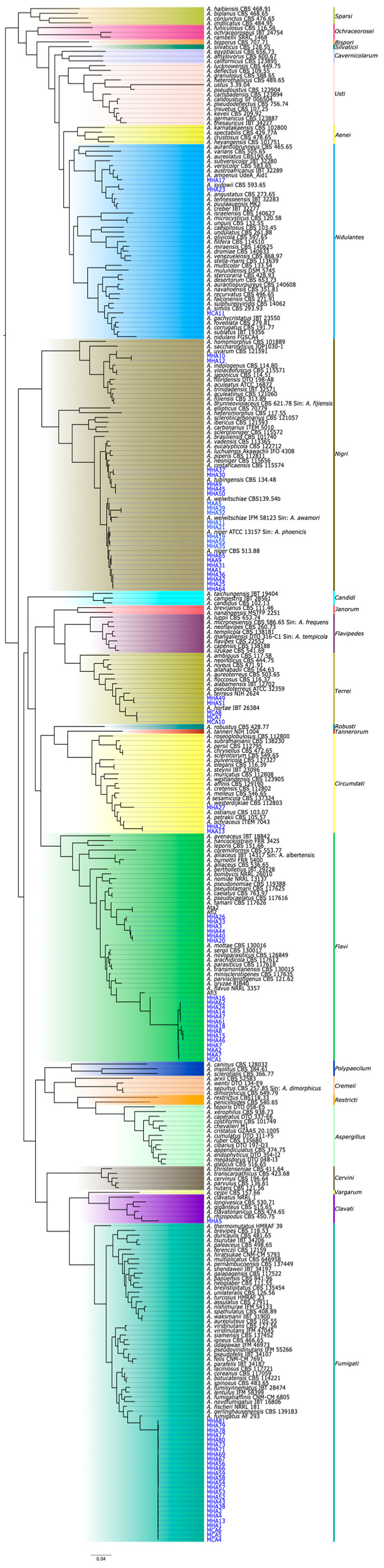
The species tree, created using OrthoFinder, illustrates the relationships of the species belonging to the genus *Aspergillus*. The species used as references are in black, the isolates from this study are represented in blue and the areas highlighted in colors refer to the taxonomic sections.

The species tree generated by the OrthoFinder program was employed to classify the isolates at the species level and to resolve any ambiguities that arose using the BLASTn tool. Consequently, the application of this algorithm allowed us to determine a phylogenomic relationship built from 323 proteomes, of which 81 were obtained in this study through *ab initio* prediction. The resulting species tree is illustrated in Figure 2.

The proteomes used were grouped into 25 sections, leaving the *Petersoniorum* and *Raperorum* sections without representatives, due to the unavailability of reference proteomes in the databases. The *Restricti* section (Figure 2, highlighted in orange) was not correctly separated, since it formed a monophyletic clade with the *Aspergillus* section (Figure 2, highlighted in turquoise). Additionally, the two species used as references for this section were paraphyletic between them.

The phylogenomic reconstruction allowed us to accurately identify all the isolates at the species level, resolving the ambiguity of the isolates in the *Flavi* and *Nigri* sections. The distribution of species showed 32% of the isolates corresponded to *A. fumigatus*, followed by *A. flavus* with 17.3% and *A. niger* with 14.8%. A lower percentage were the species *A. tamarii*, *A. tubingensis*, *A. welwitschiae* and *A. hortae*, represented by 7.4%, 6.2%, 4.9% and 3.7% of the isolates, respectively. *A. uvarum*, *A. terreus* and *A. ochraceus* followed with 2.5% each. In addition, only one representative per species of *A. spinulosporus*, *A. sydowii*, *A. westerdijkiae*, *A. amoenus* and *A. rhizopodus* was identified (Figure 1). This is the first report of these isolated species from our region and highlights the presence of rare species such as *A. hortae*, *A. uvarum*, *A. spinulosporus*, *A. sydowii*, *A. westerdijkiae*, *A. amoenus* and *A. rhizopodus*.

### 3.4. Presence of Orthologs of Allergens in the Genomes of Isolates

A list of allergens and their respective annotation codes in the reference proteomes was constructed for *A. fumigatus* Af293, *A. niger* CBS 513.88, *A. oryzae* RIB40, *A. terreus* NIH2624, *A. flavus* NRRL 3357 and *A. nidulans* FGSC A4 (Table S7) based on the genes and proteins of the officially recognized allergens that are reported in the literature and in the official allergen databases. These were used to filter the allergens of interest in the complete matrix, which yielded a total of 52,976 orthogroups. Subsequently, the orthogroup in which the recognized allergenic protein code was present was selected, and we focused on the protein codes related to the selected orthogroup for each annotated proteome of each of the isolates from this study. A total of 29 allergens were evaluated. 

In this work, we constructed a distribution map to determine the presence or absence of the 29 genes orthologous to allergens in the genomes of the 15 isolates under evaluation (Figure S4). The officially recognized allergen genes for *A. fumigatus*, *A. flavus*, *A. niger*, *A. versicolor* and *A. terreus* were present in the genomes of the evaluated species in this study, confirming that our analyses did show the expected results. Therefore, their presence was not considered during the description of the results. The orthogroup analysis denoted a broad presence of orthologous genes in the genomes and in the predicted proteome for most of the isolates. Except for the Asp f 1, Asp f 2 and Asp f 5 genes, the remaining allergenic genes contained orthologous genes in all the evaluated species. The Asp f 1 orthologs were limited to the *Clavati* and *Terrei* sections, while the Asp f 2 orthologs were present in all the analyzed sections, except for *Nigri*. The Asp f 5 orthologs, on the other hand, were found in all the analyzed sections, except for *Nidulantes*. 

The OrthoFinder analysis also showed more than one ortholog gene in some proteomes. This event was observed in the following orthologs: Asp f 3, Asp f 4, Asp f 5, Asp f 6, Asp f 9, Asp f 11, Asp f 17, Asp f 27, Asp f 28, Asp f 29, Asp o 21, Asp n 14 and Asp n 25. This characteristic was also present in the reference proteomes. In these cases, a basic phylogeny was performed with all the orthologs to the protein to establish evolutionary relationships, determine the sequence closest to allergen and thus, to finally establish the percentage of homology. Taking this into consideration, it was possible to determine, based on the homology comparison which proteins within the same orthogroup were allergens and which were of a different nature, belonging to other types of proteins in the annotated proteome of the evaluated species. Furthermore, the algorithm defined recognized allergens as orthologous (were in the same orthogroups), such as Asp f 11 and Asp f 27; Asp f 28 and Asp f 29; Asp f 18 and Asp n 18; and Asp o 13, Asp fl 13 and Asp v 13.

### 3.5. Percentage of Identity of Allergen Orthologs 

To analyze the degree of homology between the allergens described in the databases (Table S7) and the orthologs from this study, we determined the identity percentage. In the case of the orthogroups with several assigned orthologs, a single gene was selected based on the criteria of best homology and phylogenetic closeness. Figure S5 represents the percentages of identity of the orthologous genes.

We observed that several allergen orthologs presented >70% identity in all the analyzed proteomes. Among these were peroxisomal protein (Asp f 3), 60S acidic ribosomal protein (Asp f 8), molecular chaperone (Asp f 12), vacuolar serine protease (Asp f 18), enolase (Asp f 22), ribosomal protein 60 S-L3 (Asp f 23), thioredoxin (Asp f 28) and triose phosphate isomerase (Asp t 36). In general, most of the orthologous genes exhibited identity percentages greater than 50% (except for the orthologs to Asp f 1 of *A. terreus* and *A. hortae*, Asp f 7 of *A. flavus*, *A. tamarii*, *A. welwitschiae*, *A. uvarum*, *A. hortae*,and *A. westerdijkiae*, and the Asp f 17 orthologs in the species *A. amoenus* and *A. sydowii*).

The present study focuses on the evaluation of three allergens: Asp f 1, Asp f 3 and Asp f 22. Asp f 1 is an allergen that is restricted to *A. fumigatus*, and it was selected due to its wide use in the diagnosis of allergies and because it was identified in three other species. Asp f 3 is of importance in allergic diagnosis and was present in all the species evaluated. Asp f 22 is a conserved protein in our isolates and several genera of fungi. The orthogroup for Asp f 1 (XP_748109.1) was constituted by a single orthologous gene, which was exclusively detected in the species *A. fumigatus*, *A. rhizopodus*, *A. hortae* and *A. terreus*. The identity percentages for these last three species were as follows: 83.6%, 46.3% and 45%, respectively.

For Asp f 3 (XP_747849.1), two genes within the same orthogroup for each of the analyzed proteomes were obtained. An NCBI search was performed for the two protein codes that relate them to the reference species of *A. fumigatus* Af293. One of the codes corresponds to an officially recognized allergen and the other is a “putative allergen”. Consequently, a filter of the sequences with greater homology to the code of the recognized allergen in *A. fumigatus* Asp f 3 was carried out, and we observed high percentages of homology (>80%). The orthologous genes of *A. niger*, *A. welwitschiae*, *A. terreus*, *A. hortae* and *A. rhizopodus* presented higher percentages of identity (>90.4%).

The orthogroup for Asp f 22 was made up of an orthologous gene for each organism evaluated. The enolase gene was highly conserved across all the species analyzed, exhibiting identity percentages between 93 and 96%.

### 3.6. Gene Expression of Asp f 1, Asp f 3 and Asp f 22

The expression of the three orthologous allergens Asp f 1, Asp f 3 and Asp f 22 was evaluated in 15 isolates of different *Aspergillus* species. As previously described, the genetic expression of these allergens was evaluated by RT-PCR and was observed in some of the *Aspergillus* species. This finding was corroborated in *A. fumigatus*, where the allergens have been previously described [23]. The presence of 18S rRNA, as the endogenous standard, was observed in all the evaluated isolates, ensuring the cDNA quality. The transcription of Asp f 1 was evaluated exclusively in the species containing the orthologous gene in the genomic analyses, while the other two genes were evaluated in all the species. Under the conditions mentioned in the methodology, the Asp f 1 transcript was not identified in *A. hortae*. The species *A. terreus*, *A. hortae*, *A. spinulosporus*, *A. niger*, *A. tubingensis*, *A. welwitschiae* and *A. uvarum* did not have the transcript for Asp f 3. Finally, *A. niger*, *A. tubingensis*, *A. welwitschiae* and *A. uvarum* lacked Asp f 22 RNA (Table 1 and Figure S6).

## 4. Discussion

This work allowed the accurate identification of the species within the genus *Aspergillus* that were collected from different intra- and extra-hospital environments and from individuals diagnosed with aspergillosis in Medellín, Colombia. These isolates were available in the strain collection MicroCIB223. This is the first report in our country that combined whole genome sequencing with a distinct pipeline for species-level identification. In addition to their classification, this work led to the identification of candidate orthologous allergen genes in species that had not previously been studied using this approach. Finally, the presence of the transcript was evaluated, as this is a fundamental parameter for the understanding of the allergenic biology of non-*fumigatus* species.

A species-level identification was achieved by a homology search using the BLASTn algorithm with the four markers commonly used in *Aspergillus* spp. (Figure 1), resulting in a species-level resolution of 64.2%. The remaining percentage of the isolates contained identical sequences (100%) with species that make up the *Flavi* or *Nigri* sections. This was due to the high homology between the species of these sections, which has been previously documented. Additionally, the lack of a phylogenetic signal for the markers used to differentiate very similar species, despite their efficacy in this regard [42,43,44,45], may have contributed to this outcome. This suggests that the use of these four sequences (ITS, cmdA, benA and rpb2) is insufficient for resolving the taxonomic classification in some species of this genus of fungus, particularly those belonging to the *Flavi* or *Nigri* sections.

To address the challenge, this work employed the complete genome sequencing and the proteome prediction of the *Aspergillus* isolates. Using this strategy, the ambiguity in the classification of the problematic and unclassified species belonging to the sections *Flavi* and *Nigri* was effectively addressed, thereby facilitating the identification of the isolates at the species level (Figure 1 and Figure 2). These findings support the hypothesis that while the use of multiple markers is essential for differentiating between very similar species, using whole genome sequencing offers superior resolution at the species level, particularly in fungal genera that comprise many species, such as *Aspergillus*. Similar results were reported in a comparative genomics study that included 23 species from the *Flavi* section where, using a strategy similar to the one employed in the present study, *A. flavus* was also differentiated from *A. oryzae* [15]. Furthermore, in another study, a phylogenomic tree of 32 species from the *Nigri* section was described and manages to separate the species of *A. niger* from *A. welwitschiae* [46].

A significant challenge in phylogenetic reconstruction based on predicted proteomes is the limited number of reference proteomes available for many fungal species [47,48]. Similarly, one of the challenges that WGS must address is the establishment of effective, standardized and accredited bioinformatics protocols or channels [49]. Moreover, proposing agile and precise data analysis methodologies that can be implemented in laboratories with different levels of complexity is of increasing value. For this reason, in this work we propose a genomic workflow that describes the methodology and data that could be used for genome pre-assembly, assembly, annotation and barcode identification. Furthermore, this work presents a genomic workflow for the analysis of orthologous genes, with the objective of identifying allergens in species of the *Aspergillus* genus.

Regarding the allergenic potential of *Aspergillus* spp., it is noteworthy that extensive research has been conducted from the perspective of the host, elucidating the production of anti-*Aspergillus* antibodies [50,51,52,53]. However, little is known about the biology of the fungus itself as a producer of allergenic particles. Although there are 38 recognized allergens from this fungal genus, the list is far from complete, since the research on these molecules has revolved around the species *A. fumigatus*, but for other species of this genus the knowledge is limited or unknown, considering that are 446 recognized species [9]. Therefore, the present study evaluated the presence of 29 allergens in 15 different species of *Aspergillus* (Table S7 and Figure S4). It is important to highlight that these species were classified using established molecular markers and complete genome sequencing, which we believe represents a significant contribution to this field.

The identification of allergen orthologs represents a pivotal step in the recognition of potential allergens present in other species and in the characterization of possible proteins that could cross-react in the serological tests utilized in the diagnosis of allergies [54]. Using this approach, Bowyer et al. [23] employed the BLAST tool and described the presence of 16 *A. fumigatus* allergen homologous genes in *A. nidulans* and *A. oryzae*.

In this study, we implemented a process to establish the presence of a candidate orthologous gene (Figure S4) using annotated proteomes. The presence of these genes was then determined in isolates from 15 different species of the genus *Aspergillus*, and we subsequently determined the percentage of homology for the genes most closely related to the allergen of interest (Figure S5). The latter point is based on the fact that the prediction of the function of an orthologous gene must be supported by existing homology [55]. To qualify a protein as a candidate allergen, it must demonstrate >50% identity with a known allergen at the amino acid level [22]. Conversely, cross-reactivity can occur in proteins with identity levels above 50%, with a greater propensity at levels exceeding 70% [56].

As a proof of concept, the results of this genomic comparison indicate that the identified orthologs for the 29 allergens evaluated in this study may be potential allergenic proteins or may have the capacity to cross-react in serological tests. However, this hypothesis must be confirmed with new assays, such as immunoproteomic analysis. This is a significant consideration in the context of diagnostic studies of allergic entities, as it is imperative to ascertain whether an allergen is limited to a particular species or genus. Moreover, a specific allergen may possess multiple homologues in other species, and the protein in question is merely a cross-reactive member of a large family of genes present in different species [23].

In this study, we describe the presence of genes orthologous to allergens present in *Aspergillus* species that currently lack officially described allergens recognized by the WHO/IUIS committee. This includes the following species: *A. rhizopodus*, *A. welwitschiae*, *A. tubingensis*, *A. uvarum*, *A. tamarii*, *A. sydowii*, *A. amoenus*, *A. hortae*, *A. ochraceus*, *A. westerdijkiae* and *A. spinulosporus*. However, the Allergome page consolidates possible “in silico-generated” allergens for *A. rhizopodus*, *A. welwitschiae*, *A. ochraceus* and *A. sydowii* but lacks the information to validate their allergenicity [57]. Furthermore, the binding capacity of the proteins to immunoglobulin IgE for *A. tamarii* [58] and *A. ochraceus* [59] has been verified, and the proteins which could be translated by the genes were identified in this study.

To gain insight into the allergens present in the various *Aspergillus* species under investigation, we employed an expression analysis of the Asp f 1, Asp f 3 and Asp f 22 genes. Our findings indicate that most of the species that possess these genes within their genome exhibit active transcription processes for these allergens (Table 1). However, some species, including *A. terreus*, *A. hortae*, *A. spinulosporus*, *A. niger*, *A. tubingensis*, *A. welwitschiae* and *A. uvarum*, do not express some of the evaluated genes. This could be attributed to the loss of the transcription ability of these genes over evolutionary time, resulting in the formation of pseudogenes within the genomes of these species [60]. Another hypothesis is that the evaluated conditions were not favorable for the transcription of these genes. According to previous studies, the allergenic expression of the gene in *A. fumigatus* is influenced by the oxidative stress caused by hydrogen peroxide [24] and by environmental conditions such as temperature and the concentration of carbon dioxide [61,62]. The determination of allergen gene expression in *Aspergillus* spp. has only been evaluated for the *fumigatus* species. However, no studies in this area are available for the other species [24,61,62].

In this study, we present the genome sequences of *Aspergillus* isolates with a high species diversity from our geographical area (Colombian isolates), which exhibit a high prevalence of allergic diseases. We also introduce a novel approach based on whole genome sequencing to delve into the knowledge of the evolution of allergenic capacity. While the sequence homology to existing allergens is a useful tool, it is imperfect in predicting whether an unknown protein may be allergenic or cross-reactive [22]. Our findings on the establishment of homology represent a preliminary step in the process of assigning these functions, and they pave the way for future research endeavors. These include, but are not limited to, the following procedures: (i) the identification of common epitopes, (ii) an evaluation of messenger RNA expression, (iii) determination of protein translation, (iv) the verification of the binding capacity to serum IgE and finally (v) the evaluation of cross-reactivity and the induction of degranulation in basophils and mast cells. These steps are crucial for advancing our understanding of the biology and synthesis of allergens and their impact on human health.

The limitations of this study focus on various methodological and technical aspects that could influence the interpretation and the scope of the results. Firstly, the taxonomic resolution of certain genetic markers (ITS, cmdA, benA and rpb2) was insufficient to differentiate species with high homology, highlighting the need for additional markers. Additionally, the phylogenetic reconstruction based on predicted proteomes was constrained by the limited availability of reference proteomes for certain species, restricting the scope of evolutionary comparisons. Furthermore, the non-detection of allergen transcripts in some species might be attributed to the evolutionary loss of transcriptional capacity or suboptimal experimental conditions. Lastly, although orthologs of allergens were identified in understudied species, their allergenic function and cross-reactive potential were not confirmed, requiring immunoproteomic analyses in future research. These limitations underscore the importance of optimizing the used methodologies and expanding the available genomic resources to achieve a more robust characterization of the *Aspergillus* genus.

## 5. Conclusions

Whole genome sequencing is a valuable tool for species-level genomic identification. This has been demonstrated in this work by identifying a great diversity of species from the *Aspergillus* genus in our geographic area that have never been reported before. Furthermore, this tool enabled the identification of the allergenic potential of various *Aspergillus* species that had not previously been studied using this approach.

Our findings contribute to the understanding of the biology of this fungus and to the identification of novel proteins potentially implicated in the onset of allergic responses during their interaction with the human host. This could facilitate the development of more efficacious therapeutic strategies, enabling the future selection of molecules with diagnostic and immunotherapy applications in allergic aspergillosis.

## Figures and Tables

**Table 1:** Presence of Asp f 1, Asp f 3 and Asp f 22 RNA in the 15 species evaluated.

Specie	Allergen		
	Asp f 1	Asp f 3	Asp f 22
*A. fumigatus*	+	+	+
*A. rhizopodus*	+	+	+
*A. hortae*	−	−	+
*A. terreus*	+	−	+
*A. flavus*	UND	+	+
*A. tamarii*	UND	+	+
*A. niger*	UND	−	−
*A. tubingensis*	UND	−	−
*A. welwitschiae*	UND	−	−
*A. uvarum*	UND	−	−
*A. westerdijkiae*	UND	+	+
*A. ochraceus*	UND	+	+
*A. spinulosporus*	UND	−	+
*A. sydowii*	UND	+	+
*A. amoenus*	UND	+	+

UND: Undetermined, +: RNA present, −: RNA absent.

## Data Availability

The original contributions presented in the study are included in the article/Supplementary Material, further inquiries can be directed to the corresponding author.

## Supplementary Materials

The following supporting information can be downloaded at: https://www.mdpi.com/article/10.3390/jof11020098/s1, Table S1: Description of the isolates employed in this study. Table S2: Description of the isolates employed in expression assays. Table S3: Accession numbers of cmdA, benA and rpb2 genes for *Flavi* and *Nigri* sections. Table S4: The reference proteomes of *Aspergillus* spp. utilized in the OrthoFinder program. Table S5: List of primer sequences used in RT-PCR and their concentrations per reaction. Table S6: Summary parameters obtained from assembly, genomic identification, annotation and orthology statistics. Table S7: Officially recognized allergens in *Aspergillus* species by WHO/IUIS Allergen Nomenclature Sub-Committee. Figure S1: The genomics workflow. Figure S2: Phylogenetic tree from a set of concatenated nucleotide sequences (cmdA, benA and rpb2 regions) using ML analysis showing the relationship of species from the *Flavi* section. Figure S3: Phylogenetic tree from a set of concatenated nucleotide sequences (cmdA, benA and rpb2 regions) using ML analysis showing the relationship of species from the *Nigri* section. Figure S4: Distribution map of orthologous genes within the genomes of evaluated isolates. Figure S5: Heat map showing the percentage identity of predicted orthologs to proteins and to officially recognized fungal allergens. Figure S6: RT-PCR analysis of allergens in *A. fumigatus* and non-*fumigatus* species.
