# Supplementary material for: Species Identification and Orthologous Allergen Prediction and Expression in the Genus Aspergillus
Source: J Fungi (Basel). 2025 Jan 27;11(2):98. doi: 10.3390/jof11020098 (PMC11856533; doi:10.3390/jof11020098)
Supplement: Supplementary file 1 [file jof-11-00098-s001.zip › Table S1.pdf]

Table 1S. Description of the isolates employed in this study.

Isolate Code	Source	Morphological identification to section level
MHA-1	Intrahospital Air	*Fumigati*
MHA-2	Intrahospital Air	*Fumigati*
MHA-3	Intrahospital Air	*Flavi*
MHA-4	Intrahospital Air	*Fumigati*
MHA-5	Intrahospital Air	*Clavati*
MHA-7	Intrahospital Air	*Flavi*
MHA-8	Intrahospital Air	*Flavi*
MHA-9	Intrahospital Air	*Nigri*
MHA-10	Intrahospital Air	*Nigri*
MHA-11	Intrahospital Air	*Nigri*
MHA-12	Intrahospital Air	*Nigri*
MHA-13	Intrahospital Air	*Fumigati*
MHA-14	Intrahospital Air	*Flavi*
MHA-15	Intrahospital Air	*Flavi*
MHA-16	Intrahospital Air	*Flavi*
MHA-17	Intrahospital Air	*Nidulantes*
MHA-18	Intrahospital Air	*Flavi*
MHA-19	Intrahospital Air	*Nigri*
MHA-20	Intrahospital Air	*Flavi*
MHA-21	Intrahospital Air	*Nigri*
MHA-22	Intrahospital Air	*Circumdati*
MHA-23	Intrahospital Air	*Nidulantes*
MHA-24	Intrahospital Air	*Flavi*
MHA-25	Intrahospital Air	*Nigri*
MHA-26	Intrahospital Air	*Flavi*
MHA-27	Intrahospital Air	*Circumdati*
MHA-30	Intrahospital Air	*Nigri*
MHA-31	Intrahospital Air	*Nigri*
MHA-32	Intrahospital Air	*Nigri*
MHA-33	Intrahospital Air	*Flavi*
MHA-35	Intrahospital Air	*Nigri*
MHA-36	Intrahospital Air	*Nigri*
MHA-37	Intrahospital Air	*Nigri*
MHA-38	Intrahospital Air	*Fumigati*
MHA-39	Intrahospital Air	*Nigri*
MHA-40	Intrahospital Air	*Flavi*
MHA-42	Intrahospital Air	*Nigri*

MHA-43	Intrahospital Air	*Fumigati*
MHA-44	Intrahospital Air	*Flavi*
MHA-45	Intrahospital Air	*Nigri*
MHA-46	Intrahospital Air	*Flavi*
MHA-47	Intrahospital Air	*Flavi*
MHA-49	Intrahospital Air	*Terrei*
MHA-50	Intrahospital Air	*Nigri*
MHA-51	Intrahospital Air	*Terrei*
MHA-52	Intrahospital Air	*Fumigati*
MHA-53	Intrahospital Air	*Fumigati*
MHA-54	Intrahospital Air	*Fumigati*
MHA-55	Intrahospital Air	*Nigri*
MHA-56	Intrahospital Air	*Fumigati*
MHA-57	Intrahospital Air	*Fumigati*
MHA-58	Intrahospital Air	*Fumigati*
MHA-59	Intrahospital Air	*Fumigati*
MHA-61	Intrahospital Air	*Flavi*
MHA-62	Intrahospital Air	*Flavi*
MHA-64	Intrahospital Air	*Nigri*
MHA-65	Intrahospital Air	*Nigri*
MHA-66	Intrahospital Air	*Fumigati*
MHA-67	Intrahospital Air	*Fumigati*
MHA-69	Intrahospital Air	*Fumigati*
MHA-71	Intrahospital Air	*Fumigati*
MHA-73	Intrahospital Air	*Fumigati*
MHA-77	Intrahospital Air	*Fumigati*
MHA-78	Intrahospital Air	*Fumigati*
MHA-79	Intrahospital Air	*Fumigati*
MHA-80	Intrahospital Air	*Fumigati*
MHA-81	Clinical	*Fumigati*
MCA-1	Clinical	*Flavi*
MCA-4	Clinical	*Fumigati*
MCA-5	Clinical	*Fumigati*
MCA-6	Clinical	*Fumigati*
MCA-7	Clinical	*Terrei*
MCA-8	Clinical	*Terrei*
MCA-10	Clinical	*Terrei*
MCA-11	Clinical	*Nidulantes*
MAA-1	Extra hospital air	*Nigri*
MAA-2	Extra hospital air	*Flavi*
MAA-5	Extra hospital air	*Nigri*

MAA-7	Extra hospital air	*Flavi*
MAA-9	Extra hospital air	*Nigri*
MAA-13	Extra hospital air	*Circumdati*
