# Supplementary material for: Species Identification and Orthologous Allergen Prediction and Expression in the Genus Aspergillus
Source: J Fungi (Basel). 2025 Jan 27;11(2):98. doi: 10.3390/jof11020098 (PMC11856533; doi:10.3390/jof11020098)
Supplement: Supplementary file 1 [file jof-11-00098-s001.zip › Table S2..pdf]

Table 2S. Description of the isolates employed in expression assays.

Isolate Code	Source	Identification to specie level
MHA-1	Intrahospital Air	*A. fumigatus*
MHA-3	Intrahospital Air	*A. tamarii*
MHA-5	Intrahospital Air	*A. rhizopodus*
MHA-9	Intrahospital Air	*A. tubingensis*
MHA-12	Intrahospital Air	*A. uvarum*
MHA-17	Intrahospital Air	*A. amoenus*
MHA-22	Intrahospital Air	*A. ochraceus*
MHA-23	Intrahospital Air	*A. sydowii*
MHA-27	Intrahospital Air	*A. westerdijkiae*
MHA-32	Intrahospital Air	*A. welwitschiae*
MHA-49	Intrahospital Air	*A. terreus*
MCA-7	Clinical	*A. hortae*
MCA-11	Clinical	*A. spinulosporus*
MAA-2	Extra hospital air	*A. flavus*
MAA-9	Extra hospital air	*A. niger*
