# Supplementary material for: Species Identification and Orthologous Allergen Prediction and Expression in the Genus Aspergillus
Source: J Fungi (Basel). 2025 Jan 27;11(2):98. doi: 10.3390/jof11020098 (PMC11856533; doi:10.3390/jof11020098)
Supplement: Supplementary file 1 [file jof-11-00098-s001.zip › Table S3.pdf]

Table S3. Accession numbers of cmdA, benA and rpb2 genes for *Flavi* and *Nigri* sections.

***Flavi* Section**

Specie	Strain code	GenBank Accession numbers		
		benA	cmdA	rpb2
A. aflatoxiformans	CBS 143679 = DTO 228-G2T = IBT 32085	MG517706	MG518076	MG517897
	DTO 087-A2	MG517652	MG517990	MG517840
	DTO 228-G1 = IBT 32079	MG517705	MG518075	MG517896
	DTO 228-G3 = IBT 32086 = CBS 135587	MG517707	MG518077	MG517898
A. alliaceus	CBS 536.65NT = DTO 046-B1 = NRRL 315 = IMI 051982 = QM 1885 = ATCC 10760 = WB 315 = Thom 4656 = IBT 13377 = CCF 5607	EF661465	EF661534	MG517825
	CBS 143682 = DTO 326-D5 = S757 = CCF 5416 = IBT 33356	MG517764	MG518134	MG517955
	CBS 511.69 = DTO 368-C3 = IBT 13379 = CCF 5682	MG517786	MG518156	MG517976
	CBS 542.65 = DTO 034-A9 = DTO 203-B1 = NRRL 4181 = ATCC 16891 = IBT 13378 = IMI 116711 = QM 1892 = WB 4181 = JH Warcup SA 117	EF661466	EF661536	EU021644
	DTO 368-C4 = IMI 226007 = IBT 14130 = CCF 5680	MG517787	MG518157	MG517977
	IBT 21770	MG517790	MG518161	MG517980
A. arachidicola	CBS 117610T = DTO 009-G3 = IBT 25020	EF203158	EF202049	MG517802
	DTO 228-H9	MG517721	MG518091	MG517912
A. aspearensis	CBS 143672T = DTO 203-D9 = CCTU758 = IBT 32590 = IBT 34544	MG517669	MG518040	MG517857
	DTO 203-D4 = CBS 143671 = CCTU753 = IBT 34543	MG517667	MG518038	MG517855
A. austwickii	CBS 143677T = DTO 228-F7 = IBT 32590 = IBT 32076	MG517702	MG518072	MG517893

	CBS 135406 = DTO 228-G8 = IBT 32091	MG517712	MG518082	MG517903
	DTO 228-F9 = IBT 32078	MG517704	MG518074	MG517895
A. avenaceus	CBS 109.46T = DTO 009-H6 = DTO 006-A2 = NRRL 517 = ATCC 16861 = IMI 016140 = LCP 89.2592 = LSHB BB 155 = QM 6741 = WB 317 = IBT 4376 = IBT 4555	FJ491481	FJ491496	JN121424
	CBS 102.45 = NCTC 6548	FJ491480	FJ491495	–
A. bertholletius	CBS 143687 = DTO 223-D3 = IBT 29228 = CCT 7615T = ITAL 270/06	MG517689	JX198674	MG517880
A. caelatus	CBS 763.97T = DTO 046-A8 = NRRL 25528 = IBT 21091	MG517640	MG518018	MG517823
	DTO 276-I2	MG517738	MG518108	MG517929
	DTO 285-H9	MG517751	MG518121	MG517942
	NRRL 25566 = IBT 29770 = DTO 073-B7	MG517651	MG518025	MG517839
	NRRL 26100	EF661471	EF661523	EF661437
A. cerealis	CBS 143674T = DTO 228-E7 = IBT 32067	MG517693	MG518063	MG517884
	DTO 228-E6 = IBT 32076	MG517692	MG518062	MG517883
	DTO 228-F1 = IBT 32070	MG517696	MG518066	MG517887
A. coremiiformis	CBS 553.77T = DTO 046-A3 = ATCC 38576 = IHEM 4503 = IMI 223069 = NRRL 13603 = NRRL 13756 = IBT 3822 = IBT 13506 = IBT 21944	FJ491482	FJ491488	JN121533
A. flavus	CBS 100927T = NRRL 1957 = ATCC 16883 = CBS 569.65 = IMI 124930 = IBT 3605 = IBT 3610	EF661485	EF661508	EF661440
	CBS 110.55 = DTO 046-A1 = ATCC 12073 = NRRL 4743 = IMUR 236 = QM 6951 = WB 4743 = IBT 3819	EF203135	MG518005	MG517821
	CBS 117638 = DTO 009-G1	MG517619	MG518011	MG517801
	CBS 119368 = DTO 011-I2 = KACC 41730	MG517630	MG518002	MG517813

	CBS 120.51 = DTO 046-A4 = ATCC 16859 = IFO 8135 = IMI 045644 = LCP 56.1517 = LSHB BB213 = NRRL 2097 = NRRL A-2022 = QM 6871 = WB 2097 = IBT 3636	MG517639	MG518012	MG517822
	CBS 133263 = DTO 215-E9	JX627689	JX627693	MG517868
	CBS 485.65 = DTO 046-B7 = ATCC 16870 = IFO 5324 = IMI 124932 = LCP 89.3556 = NRRL 4818 = WB 4818 = IBT 3641 = IBT 3657	MG517643	MG518014	MG517828
	CBS 501.65 = DTO 046-B5 = ATCC 16862 = IMI 044882 = NRRL 4998 = WB 4998 = IBT 4378 = IBT 4402	MG517642	MG518015	MG517827
	CBS 542.69 = DTO 046-B4 = IMI 141553 = NRRL 3751 = GKC 1421(1) = IBT 3649	MG517641	MG518016	MG517826
	CBS 574.65 = DTO 303-C3 = ATCC 1010 = IMI 016142 = IMI 124935 = NRRL 506 = NRRL 1653	JN185446	JN185447	JN185449
	NRRL 20521	EF661492	EF661514	EF661447
	NRRL 3518 = NRRL A-14304	EF661487	EF661510	EF661442
A. lanosus	CBS 650.74T = IMI 130727 = QM 9183 = IBT 33634 =NRRL 3648	MG517633	MG518017	EU021642
A. leporis	CBS 151.66T = IBT 3609 = DTO 199-B2 = CBS 129302 = RMF 99 = WB 5188 = ATCC 16490 = LCP 89.2583 = NRRL 3216	MG517662	MG518033	MG517850
	CBS 125914 = DTO 195-C3 = R1251	MG517660	MG518031	MG517848
	CBS 129235 = DTO 303-C5	MG517760	MG518130	MG517951
	CBS 129330 = RMF 7757 = DTO 202-A2	MG517664	MG518035	MG517852
A. luteovirescens	CBS 620.95T = DTO 010-H1	MG517625	MG517998	MG517808
A. minisclerotigenes	CBS 117635T = DTO 009-F7 = IBT 25032	EF203148	MG518009	MG517799
	CBS 117633 = DTO 009-F5	EF203153	MG518007	MG517797
	CBS 117634 = DTO 009-F6 = IBT 27197	MG517617	MG518008	MG517798

	DTO 045-I9 = NRRL A-11611 = NRRL 6444 = IBT 3840	MG517638	MG518024	MG517820
	DTO 228-H5 = IBT 24629	MG517718	MG518088	MG517909
A. mottae	CBS 130016T = DTO 223-C8 = IBT 32309 = MUM 10.231	MG517687	MG518058	MG517878
	MUM 10.233	HM803090	HM803013	HM802982
A. neoalliaceus	CBS 143681T = DTO 326-D3 = S765 = CCF 5433 = IBT 33110 = IBT 33353	MG517763	MG518133	MG517954
	DTO 326-D6 = S768 = CCF 5414 = IBT 33111 = IBT 33357	MG517765	MG518135	MG517956
	DTO 326-D7 = B6 = CCF 5408 = IBT 32726	MG517766	MG518136	MG517957
A. nomius	CBS 260.88T = NRRL 13137 = IBT 3656 = IBT4966 = FDA M93	EF661494	EF661531	EF661456
	DTO 161-F2	MG517657	MG518027	MG517845
	DTO 226-I5	MG517690	MG518060	MG517881
	DTO 243-E8	MG517722	MG518092	MG517913
	DTO 247-F9	MG517723	MG518093	MG517914
	DTO 318-F4	MG517761	MG518131	MG517952
A. novoparasiticus	CBS 126849T = DTO 223-C3 = DTO 223-C4 = FMR 10121 = LEMI 250 = IBT 32311	MG517684	MG518055	MG517875
	CBS 126850 = DTO 223-C5 = FMR 10158 = LEMI 149 IOP = IBT 32312	MG517686	MG518057	MG517877
A. oryzae	CBS 102.07T = CBS 110.47 = CBS 100925 = ATCC 1011 = ATCC 12891 = ATCC 4814 = ATCC 7651 = ATCC 9102 = CECT 2094 = IFO 4075 = IFO 5375 = IMI 016266ii = IMI 016266 = IMI 044242 = LSHBA c.19 = NCTC 598 = NRRL 447 = NRRL 692 = QM 6735 = Thom 113 = WB 447 = IBT 21451	EF661483	EF661506	EF661438

A. parasiticus	CBS 100926T = NRRL 502 = ATCC 1018 = ATCC 6474 = ATCC 7865 = IMI 015957 = IMI 015957ii = IMI 015597iv = IMI 015957vi = IMI 015957vii = IMI 015957ix = NRRL 1731 = IBT 3607	EF661481	EF661516	EF661449
	CBS 138.52 = DTO 009-H4	MG517623	MG517997	MG517806
	CBS 260.67 = DTO 046-C2 = ATCC 15517 = CCM F-550 = CECT 2680 = DSM 2038 = IFO 30179 = IHEM 4387 = IMI 120920 = IMI 229041 = MUCL 31311	EF203156	MG518013	MG517830
	CBS 580.65 = DTO 046-B9 = ATCC 1014 = ATCC 16863 = IMI 016127ii = LSHB Ac22 = NCTC 974 = NRRL 424 = QM 7475 = VKM F-2041 = WB 424 = IBT 3664 = IBT 3670 = IBT 10828	MG517644	MG518030	MG517829
	CBS 822.72 = DTO 046-A9 = ATCC 22789 = IFO 30109 = IMI 089717 = RIB 4002 = TRI M 39 = IBT 4377 = IBT 4408	EF203163	MG518019	MG517824
	DTO 203-C4	MG517666	MG518037	MG517854
	DTO 258-D1	MG517726	MG518096	MG517917
	DTO 285-G9	MG517750	MG518120	MG517941
	NRRL 6433 = IBT 4375	EF661480	EF661519	EF661452
A. pipericola	CBS 143680T = DTO 228-H4 = IBT 24628	MG517717	MG518087	MG517908
A. pseudocaelatus	CBS 117616T = DTO 010-H4 = IBT 27191	MG517626	MG517995	MG517809
A. pseudonomius	CBS 119388T = DTO 009-F1 = NRRL 3353 = IBT 27864 = IBT 14897	EF661495	EF661529	EF661454
	DTO 177-G7	MG517659	MG518029	MG517847
	DTO 262-F3	MG517729	MG518099	MG517920
	NRRL 6552	EF661496	EF661528	EF661455
A. pseudotamarii	CBS 766.97T = NRRL 25517 = DTO 046-C1 = IBT 21092	EF661477	EF661521	EU021631
	CBS 765.97 = NRRL 443	EF661476	EF661520	EU021650
A. sergii	CBS 130017T = DTO 223-C9 = IBT 32292 = IBT 32293	MG517688	MG518059	MG517879

A. sojae	CBS 100928T = DTO 046-C3 = ATCC 42251 = IAM 2669 = IFO 4244 = IFO 30112 = IMI 191300 = RIB 1045 = SRRC 1126 = K. Sakaguchi SH-10-6 = IBT 21642 = IBT 32109	EF203168	EF202041	MG517831
A. subflavus	CBS 143683T = DTO 326-E8 = S778 = CCF 4957 = NRRL 66254 = IBT 34939	MG517773	MG518143	MG517964
	S843b	MG517792	MG518164	MG517983
A. tamarii	CBS 104.13T = NRRL 20818 = QM 9374 = IBT 3648	EF661474	EF661526	EU021629
	CBS 133097 = DTO 213-H5 = NRRL 4959	MG517678	MG518049	MG517866
	DTO 010-G9 = CBS 167.63 = NRRL 4680 = ATCC 15054 = IMI 172295 = QM 8903 = WB 4680 = IBT 22566	MG517624	MG518001	MG517807
	DTO 065-A4	MG517648	MG517984	MG517835
	DTO 066-A1	MG517649	MG517988	MG517836
	DTO 266-D7	MG517730	MG518100	MG517921
	DTO 364-E3	MG517781	MG518151	MG517971
	NRRL 425	EF661475	EF661524	EU021648
	NRRL 426 = DTO 010-H3 = CBS 579.65 = IBT 3681 = IBT 3826 = IBT 10827	EF661472	EF661525	EU021649
	NRRL 4911 = CBS 484.65 = IBT 3659	EF661473	EF661527	EU021651
A. togoensis	CBS 272.89 = DTO 034-C1 = NRRL 13550 = IBT 14989 = IBT 21943	FJ491477	FJ491489	JN121479
A. transmontanensis	CBS 130015T = MUM 10.214 = IBT 32313	HM803101	HM803020	HM802980
	MUM 10.205	HM803087	HM803021	HM802979
A. vandermerwei	CBS 612.78T = DTO 069-D2 = DTO 034-B5 = NRRL 5108 = IBT 13876 = CCF 5683	EF661469	EF661540	MG517838
	DTO 368-C1 = NRRL 1236 = IBT 13865 = CCF 5685	MG517784	MG518154	MG517974
	DTO 368-C2 = CBS 126709 = RMF 9585 = IBT 20468 = CCF 5681	MG517785	MG518155	MG517975

	IBT 16662	MG517788	MG518162	MG517978
	IBT 20491	MG517789	MG518163	MG517979
A. ochraceus	NRRL 398	EF661322	EF661381	EF661302
A. robustus	NRRL 6362	EU014101	EF661357	EF661033

Nigri Section

Specie	Strain code	benA	cmdA	rpb2
A. neoniger	CBS 115656 = NRRL 62634	FJ491691	FJ491700	KC796429
A. costaricensis	CBS 115574 = IBT 23401 = CECT 20579 = ITEM 7555	FJ629277	FN594545	HE984361
A. vadensis	CBS 113365 = CECT20584 = IMI 313493	AY585531	FN594560	HE984371
A. eucalypticola	CBS 122712 = IBT 29274	EU482435	EU482433	MN969070
*A. tubingensis (*Previamente: *A. acidus)*	NRRL 4875 = QM 8904 = WB 4875 = CBS 133056	EF661086	EF661151	EF661055
	NRRL 365	EF661084	EF661149	EF661053
A. luchuensis	CBS 205.80 = NBRC 4281 = KACC 46772 = IFM 47726 = RIB 2642	JX500062	JX500071	MN969081
A. piperis	CBS 112811 = IBT 24630 = IBT 26239 = NRRL 62631	FJ629303	EU163267	KC796427
*A. niger (*Previamente: *A. lacticofeatus,* y *A. phoenincis)*	CBS 554.65 = NRRL 326 = ATCC 16888 = IFO 33023 = IHEM 3415 = IMI 050566ii = IMI 50566 = JCM 10254 = QM 9270 = QM 9946 = Thom 2766 = WB326	EF661089	EF661154	EF661058
	ATCC=9029	KU897006	JF450801	
	NRRL 348	EF661091	EF661158	EF661057
	CBS 513.88	MG701893	ANI_1_1116184	

	NRRL 3	EF661088	EF661153	EF661056
A. welwitschiae (Previamente: *A. awamori)*	CBS 139.54	MN969369	KC480196	MN969100
A. brasiliensis	CBS 101740 = IMI 381727 = IBT 101740	FJ629272	FN594543	KY006765
	NRRL 26651	EF661094	EF661160	EF661064
	NRRL 35542	EF661096	EF661162	
A. sclerotioniger	CBS 115572 = IBT 22905= ITEM 7560	FJ629304	FN594557	
A. sclerotiicarbonarius	CBS 121057 = IBT 121057	EU159229	EU159235	MN969091
	CBS 121851	EU159230	EU159239	
A. carbonarius	CBS 111.26 = NRRL 369 = ATCC 1025 = IMI 16136 = LSHBA c .11 = NCTC 1325 = NRRL 1987 = QM 331 = Thom 4030.1 = WB 369	EF661099	EF661167	EF661068
	NRRL 67	EF661097	EF661165	EF661066
	NRRL 4849	EF661100	EF661168	EF661069
A. ibericus	NRRL 35644	EF661102	EF661163	EF661065
	NRRL 35645	EF661101	EF661164	
A. ellipticus	CBS 482.65 = CBS 707.79 = DTO 035-B7 = NRRL 5120 = ATCC 16876 = IMI 172283 = NRRL 20624 = QM 8886 = WB 5120	AY585530	EF661170	EF661051
A. heteromorphus	CBS 117.55 = NRRL 4747 = ATCC 12064 = IMI 172288 = QM 6954 = WB 4747	EF661103	EF661169	EF661050
A. aculeatinus	CBS 121060 = DTO 202-G5 = IBT 29077	EU159220	EU159241	HF559233
	ITEM 13553	HE984407	HE984422	HE984359
A. assiutensis	CBS 132773 = AUMC 5748			
A. trinidadensis	DTO 198-D1 = NRRL 62479 = ITEM 14821	HE984420	HE984434	HE984379
	ITEM 14829 =NRRL 62480	HE984410	HE984425	HE984373
A. brunneoviolaceus (Previamente: *A. fjiensis)*	CBS 621.78 = NRRL 4912 = IMI 312981 = WB 4912	EF661105	EF661147	EF661045
	ITEM 14784	HE984411	HE984426	HE984374
A. aculeatus (Previamente *A. kawachii)*	CBS 172.66 = NRRL 5094 = NRRL 20623 = IMI 211388 = ATCC 16872 = WB 5094	HE577806	EF661148	EF661046
	ITEM 4760 = CBS 620.78=NRRL 2053	EU982087	EF661145	EF661044
	ITEM 15927 = NRRL 359	EF661106	EF661146	EF661043

A. floridensis	DTO 198-A8 = NRRL 62478 = ITEM 14783	HE984412	HE984429	HE984376
A. japonicus (Previamente: *violaceofuscus*	CBS 114.51 = ITEM 7034	HE577804	FN594551	MN969079
	NRRL 35494		EU021690	EU021639
A. indologenus	CBS 114.80 = IBT 3679 = ITEM 7038	AY585539	AM419750	HE984366
A. uvarum	CBS 121591 = IBT 26606 = IMI 388523 = ITEM 4834	AM745751	AM745755	HE984370
	ITEM 14819	HE984421	HE984435	HE984380
	ITEM 5325	AM745753	AM745756	
A. serratalhadensis	URM 7866	LT993222	LT993223	
A. labruscus	DTO 357-D4 = ITAL 22.223 = IBT 33586	KT986014	KT986008	MN969196
	ITAL 31.311	KT986019	KT986013	
	ITAL 28.255	KT986018	KT986012	
A. saccharolyticus	CBS 127449 = IBT 28509	HM853553	HM853554	HF559235
A. homomorphus	CBS 101889 = ITEM 7556	AY820015	FN594549	MN969076
