# Supplementary material for: Species Identification and Orthologous Allergen Prediction and Expression in the Genus Aspergillus
Source: J Fungi (Basel). 2025 Jan 27;11(2):98. doi: 10.3390/jof11020098 (PMC11856533; doi:10.3390/jof11020098)
Supplement: Supplementary file 1 [file jof-11-00098-s001.zip › Table S4.pdf]

Table S4. Reference proteomes of *Aspergillus* spp. used in OrthoFinder program.

Specie	Strain	Data base
A. fumigatus	Af293	NCBI
A. oryzae	RIB40	NCBI
A. niger	CBS 513.88	NCBI
A. terreus	NIH2624	NCBI
A. luchuensis	AkawachiiIFO4308	NCBI
A. flavus	NRRL 3357	NCBI
A. nidulans	FGSC A4	NCBI
A. fisherii	NRRL 181	NCBI
A. clavatus	NRRL 1	NCBI
A. phoenicis	ATCC 13157	NCBI
A. homomorphus	CBS 101889	NCBI
A. ibericus	CBS 121593	NCBI
A. costaricaensis	CBS 115574	NCBI
A. fijiensis	CBS 313.89	NCBI
A. japonicus	CBS 114.51	NCBI
A. aculeatinus	CBS 121060	NCBI
A. piperis	CBS 112811	NCBI
A. brunneoviolaceus	CBS 621.78	NCBI
A. violaceofuscus	CBS 115571	NCBI
A. indologenus	CBS 114.80	NCBI
A. sclerotiicarbonarius	CBS 121057	NCBI
A. ellipticus	CBS 707.79	NCBI
A. uvarum	CBS 121591	NCBI
A. neoniger	CBS 115656	NCBI
A. saccharolyticus	JOP 1030-1	NCBI
A. vadensis	CBS 113365	NCBI
A. heteromorphus	CBS 117.55	NCBI
A. sclerotioniger	CBS 115572	NCBI
A. eucalypticola	CBS 122712	NCBI
A. steynii	IBT 23096	NCBI
A. campestris	IBT 28561	NCBI
A. novofumigatus	IBT 16806	NCBI

A. awamori	IFM 58123	NCBI
A. ochraceoroseus	IBT 24754	NCBI
A. ruber	CBS 135680	NCBI
A. brasiliensis	CBS 101740	NCBI
A. sydowii	CBS 593.65	NCBI
A. versicolor	CBS 583.65	NCBI
A. tubingensis	CBS 134.48	NCBI
A. glaucus	CBS 516.65	NCBI
A. parasiticus	CBS 117618	NCBI
A. aculeatus	ATCC 16872	NCBI
A. carbonarius	ITEM 5010	NCBI
A. nomiae	NRRL 13137	NCBI
A. wentii	DTO 134E9	NCBI
A. ustus	strain:3.3904	NCBI
A. creber	IBT 32277	Mycocoms
A. pseudoviridinutans	IFM 55266	NCBI
A. puulaauensis	MK2	NCBI
A. elegans	CBS 116.39	Mycocoms
A. hiratsukae	CNM-CM5793	NCBI
A. oerlinghausenensis	CBS 139183	Mycocoms
A. floridensis	DTO 198-A8	Mycocoms
A. amoenus	UdeA_Aid1	Mycocoms
A. pseudonomiae	CBS 119388	NCBI
A. caelatus	CBS 763.97	NCBI
A. bertholletiae	IBT 29228	NCBI
A. pseudocaelatus	CBS 117616	NCBI
A. coremiiformis	CBS 553.77	NCBI
A. transmontanensis	CBS 130015	NCBI
A. sergii	CBS 130017	NCBI
A. avenaceus	IBT 18842	NCBI
A. pseudotamarii	CBS 117625	NCBI
A. novoparasiticus	CBS 126849	NCBI
A. minisclerotigenes	CBS 117635	NCBI
A. alliaceus	CBS 536.65	NCBI
A. leporis	CBS 151.66	NCBI
A. tamarii	CBS117626	NCBI
A. ochraceus	ITEM 7043	Mycocoms
A. cejpii	CBS 157.66	Mycocoms

A. viridinutans	IFM 47045	NCBI
A. tanneri	NIH1004	NCBI
A. mulundensis	DSM 5745	NCBI
A. welwitschiae	CBS 139.54b	NCBI
A. unguis	CBS132.55	Mycocoms
A. pseudoterreus	ATCC 32359	Mycocoms
A. taichungensis	IBT 19404	NCBI
A. candidus	CBS 102.13	NCBI
A. arachidicola	CBS 117612	NCBI
A. thermomutatus	HMR AF 39	NCBI
A. turcosus	HMR AF 23	NCBI
A. persii	CBS 112795	Mycocoms
A. bombycis	NRRL 26010	NCBI
A. cristatus	GZAAS20.1005	Mycocoms
A. chevalieri	M1	NCBI
A. calidoustus	SF006504	NCBI
A. lentulus	IFM 58399	NCBI
A. westerdijkiae	CBS 112803	Mycocoms
A. sclerotiorum	CBS 549.65	Mycocoms
A. udagawae	IFM 46973	NCBI
A. rambellii	SRRC1468	NCBI
A. melleus	CBS 546.65	NCBI
A. nanangensis	MST-FP2251	NCBI
A. felis	CNM-CM7691	NCBI
A. burnettii	FRR 5400	NCBI
A. latus sinónimo *A. sublatus*	IBT 19356	Mycocoms
A. fumigatiaffinis	CNM-CM6805	NCBI
A. floccosus	CBS 116.37	Mycocoms
A. sclerotialis	CBS 366.77	Mycocoms
A. hancockii	FRR 3425	Mycocoms
A. affinis	CBS 129190	Mycocoms
A. alabamensis	IBT 12702	Mycocoms
A. allahabadii	CBS 164.63	Mycocoms
A. ambiguus	CBS 117.58	Mycocoms
A. amylovorus/A.cavernicola	CBS 600.67	Mycocoms
A. angustatus	CBS 273.65	Mycocoms
A. appendiculatus	CBS 374.75	Mycocoms
A. arxii	CBS 52583	Mycocoms

A. assulatus	CBS 27911	Mycocoms
A. astellatus	CBS 261.93	Mycocoms
A. aurantiobrunneus	CBS 465.65	Mycocoms
A. aurantiopurpureus	CBS 140608	Mycocoms
A. aureolatus	CBS 190.65	Mycocoms
A. aureoluteus	CBS 105.55	Mycocoms
A. aureoterreus	CBS 503.65	Mycocoms
A. austroafricanus	IBT 32289	Mycocoms
A. biplanus	CBS 468.65	Mycocoms
A. bisporus	CBS 707.71	Mycocoms
A. botucatensis	CBS 114221	Mycocoms
A. brevijanus	CBS 111.46	Mycocoms
A. brevipes	CBS 118.53	Mycocoms
A. brevistipitatus	CBS 135454	Mycocoms
A. caespitosus	CBS 103.45	Mycocoms
A. californicus	CBS 123895	Mycocoms
A. caninus	CBS 128032	Mycocoms
A. capensis	CBS 138188	Mycocoms
A. caperatus	DTO 337-E6	Mycocoms
A. carlsbadensis	CBS 123894	Mycocoms
A. cervinus	CBS 196.64	Mycocoms
A. christenseniae	CBS 411.64	Mycocoms
A. chrysellus	CBS 472.65	Mycocoms
A. cibarius	DTO 197-D3	Mycocoms
A. clavatonanicus	CBS 474.65	Mycocoms
A. conjunctus	CBS 476.65	Mycocoms
A. coreanus	CBS 117059	Mycocoms
A. corrugatus	CBS 191.77	Mycocoms
A. costiformis	CBS 101749	Mycocoms
A. flavipes	CBS 22552	Mycocoms
A. giganteus	CBS 515.65	Mycocoms
A. hortae	IBT 26384	Mycocoms
A. cretensis	CBS 112802	Mycocoms
A. crustosus	CBS 478.65	Mycocoms
A. cumulatus	DTO 311-F5	Mycocoms
A. deflectus	CBS 109.55	Mycocoms
A. desertorum	CBS 653.73	Mycocoms
A. dromiae	CBS 140633	Mycocoms

A. duricaulis	CBS 481.65	Mycocoms
A. egyptiacus	CBS 656.73	Mycocoms
A. endophyticus	DTO 354-I2	Mycocoms
A. falconensis	CBS 271.91	Mycocoms
A. ferenczii	CBS 12159	Mycocoms
A. filifera	CBS 114510	Mycocoms
A. foveolatus	CBS 279.81	Mycocoms
A. frequens	CBS 586.65	Mycocoms
A. fumisynnematus	IBT 28474	Mycocoms
A. funiculosus	CBS 116.56	Mycocoms
A. galapagensis	CBS 117522	Mycocoms
A. germanicus	CBS 123887	Mycocoms
A. granulosus	CBS 588.65	Mycocoms
A. haitiensis	CBS 468.91	Mycocoms
A. heterothallicus	CBS 489.65	Mycocoms
A. heyangensis	CBS 101751	Mycocoms
A. igneus	CBS 466.65	Mycocoms
A. iizukae	CBS 541.69	Mycocoms
A. implicatus	CBS 484.95	Mycocoms
A. insolitus	CBS 384.61	Mycocoms
A. insuetus	CBS 107.25	Mycocoms
A. israelensis	CBS 140627	Mycocoms
A. karnatakaensis	CBS 102800	Mycocoms
A. keveii	CBS 209.92	Mycocoms
A. laciniosus	CBS 117721	Mycocoms
A. lucknowensis	CBS 449.75	Mycocoms
A. luppii	CBS 653.74	Mycocoms
A. mangaliensis	DTO 316-C1	Mycocoms
A. megasporus	DTO 048-I3	Mycocoms
A. microcysticus	CBS 120.58	Mycocoms
A. miraensis	CBS 140625	Mycocoms
A. mottae	CBS 130016	Mycocoms
A. multiplicatus	CBS 646958	Mycocoms
A. neoflavipes	CBS 260.73	Mycocoms
A. neoglaber	CBS 111.55	Mycocoms
A. neoindicus	CBS 444.75	Mycocoms
A. nishimurae	IFM 54133	Mycocoms
A. niveus	CBS 471.91	Mycocoms

A. nutans	CBS 121.56	Mycocoms
A. olivicola	CBS 597.65	Mycocoms
A. ostianus	CBS 103.07	Mycocoms
A. pachycristatus	IBT 23550	Mycocoms
A. paleaceus	CBS 498.65	Mycocoms
A. papuensis	CBS 841.96	Mycocoms
A. parafelis	IBT 34187	Mycocoms
A. parvisclerotigenus	CBS 121.62	Mycocoms
A. parvulus	CBS 136.61	Mycocoms
A. penicilloides	CBS 540.65	Mycocoms
A. pernambucoensis	CBS 137449	Mycocoms
A. petrakii	CBS 105.57	Mycocoms
A. pseudodeflectus	CBS 756.74	Mycocoms
A. pseudofelis	IBT 34107	Mycocoms
A. pseudoustus	CBS 123904	Mycocoms
A. pulvericola	CBS 137327	Mycocoms
A. recurvatus	CBS 496.65	Mycocoms
A. restrictus	CBS 118.33	Mycocoms
A. rhizopodus	CBS 450.75	Mycocoms
A. robustus	CBS 428.77	Mycocoms
A. roseoglobulosus	CBS112800	Mycocoms
A. sepultus	CBS 257.85	Mycocoms
A. sesamicola	CBS 137324	Mycocoms
A. shendaweii	IBT 34197	Mycocoms
A. siamensis	CBS 137452	Mycocoms
A. silvaticus	CBS 128.55	Mycocoms
A. similis	CBS 293.93	Mycocoms
A. spathulatus	CBS 408.89	Mycocoms
A. spectabilis	CBS 429.77A	Mycocoms
A. spinosus	CBS 483.65	Mycocoms
A. stella-maris	CBS 113639	Mycocoms
A. stercorarius	CBS 428.93	Mycocoms
A. subramanianii	CBS 138230	Mycocoms
A. subversicolor	IBT 32280	Mycocoms
A. sulphureoviridis	CBS 14062	Mycocoms
A. templicola	CBS 138181	Mycocoms
A. tennesseensis	IBT 32283	Mycocoms
A. teporis	DTO058-E5	Mycocoms

A. thesauricus	IBT 34227	Mycocoms
A. transcarpathicus	CBS 423.68	Mycocoms
A. trinidadensis	IBT 32571	Mycocoms
A. tsurutae	IBT 34206	Mycocoms
A. undulatus	CBS 261.88	Mycocoms
A. unilateralis	CBS 126.56	Mycocoms
A. varians	CBS 505.65	Mycocoms
A. venezuelensis	CBS 868.97	Mycocoms
A. viridinutans	CBS 127.56	Mycocoms
A. waksmanii	IBT 31900	Mycocoms
A. westlandensis	CBS 123905	Mycocoms
A. xerophilus	CBS 938.73	Mycocoms
A. muricatus	CBS 112808	Mycocoms
A. navahoensis	CBS 351.81	Mycocoms
A. multicolor	CBS 133.54	Mycocoms
A. longivesica	CBS 530.71	Mycocoms
