# Supplementary material for: Species Identification and Orthologous Allergen Prediction and Expression in the Genus Aspergillus
Source: J Fungi (Basel). 2025 Jan 27;11(2):98. doi: 10.3390/jof11020098 (PMC11856533; doi:10.3390/jof11020098)
Supplement: Supplementary file 1 [file jof-11-00098-s001.zip › Table S5.pdf]

Table S5. List of primer sequences used to RT-PCR and its concentrations per reaction.

Gen	Target evaluated	Primers	Primer's concentration (µM)
**18 S rRNA**	Universal (15 species) *	Forward: GAAAGCATTCGCCAAGGATG	0.3
		Reverse: CTACGACGGTATCTGATCGTC	
**Asp f 1**	*A. fumigatus*	Forward: ATACAATCAAGCCAAAGCCG	0.3
		Reverse: GTAGCCGTTAGTGAACCAGT	
	A. rhizopodus	Forward: CGCCCATCAAGTTCGGAAAG	0.3
		Reverse: GGAACTCCAGCAGGTAGTGA	
	A. hortae - A. terreus	Forward: TGGTGGCATAAACGACCAT	0.3
		Reverse: TCCGCTCTGTTACCAAAGG	
**Asp f 3**	*A. fumigatus - A. flavus - A. tamarii - A. rhizopodus - A. hortae - A. terreus*	Forward: ATCCCCATCAACTACAACGC	0.2
		Reverse: TCATGACGTAGGCATCGTT	
	A. niger - A. tubingensis - A. welwitschiae -A. uvarum	Forward: AAGAAGGTCATCCTCTTCGC	0.2
		Reverse: TGACGTAGGCATCGTTGTAG	
	A. ochraceus - A. westerdijkiae	Forward: CTTGCGGTATCCCCATCAA	0.2
		Reverse: AGGATGACCTTCTTGTCGG	
	A. spinulosporus - A. sydowii - A. amoenus	Forward: AGATCACCTCCTGCGGTAT	0.2
		Reverse: AGGATGACCTTCTTGTTGGC	
**Asp f 22**	Universal (15 species) *	Forward: GAGGTCTACCAGAAGCTCAA	0.4
		Reverse: CTGAATATCGGGAGCAACAC	

**A. fumigatus, A. flavus, A. tamarii, A. rhizopodus, A. hortae, A. terreus A. niger, A. tubingensis, A. welwitschiae, A. uvarum, A. ochraceus, A. spinulosporus, A. sydowii, A. westerdijkiae* and *A. amoenus.*
