# Supplementary material for: Species Identification and Orthologous Allergen Prediction and Expression in the Genus Aspergillus
Source: J Fungi (Basel). 2025 Jan 27;11(2):98. doi: 10.3390/jof11020098 (PMC11856533; doi:10.3390/jof11020098)
Supplement: Supplementary file 1 [file jof-11-00098-s001.zip › Table S6.pdf]

Table S6. Summary parameters obtained from assembly, genomic identification, annotation and orthology statistics.

Isolate name	a. Identificación BLAST web	b. Assembly quality parameters					c. Annotation and orthology statistics	
		Total length	%GC	N50	L50	# Contigs	# Predicted gens	Percentage of genes in orthogroups (%)
MAA-1	*Nigri*	36474363	49,52	422783	26	299	12881	99.9
MAA-2	*Flavi*	38338570	47,24	683005	19	334	14042	100.0
MAA-5	*Nigri*	36827352	49,37	676150	17	374	12989	99.7
MAA-7	*Flavi*	37787574	47,31	678905	17	208	13954	99.9
MAA-9*	*Nigri*	37878637	49,47	470795	25	749	13149	99.7
MAA-13	*A. ochraceus*	36661193	49,55	361678	30	345	15146	99.9
MCA-1	*Flavi*	37934007	47,32	764788	15	261	14040	99.9
MCA-4*	*A. fumigatus*	34032820	49,82	250146	39	5240	17994	81.5
MCA-5	*A. fumigatus*	28508917	49,47	426370	24	244	11719	99.7
MCA-6	*A. fumigatus*	28617313	49,4	405201	25	219	11621	99.9
MCA-7	*A. hortae*	29879840	52,19	929943	12	176	14688	99.8
MCA-8	*A. hortae*	31186178	51,98	708511	14	235	14995	99.9
MCA-10	*A. hortae*	31926451	52,04	641520	17	388	15247	99.7
MCA-11*	*A. spinulosporus*	31431361	49,79	334113	26	1258	12840	95.4
MHA-1	*A. fumigatus*	28350389	49,51	431690	19	312	11790	99.8
MHA-2	*A. fumigatus*	28970068	49,46	492931	19	315	11872	99.5
MHA-3	*A. tamarii*	38974335	47,22	581201	22	266	13762	99.8
MHA-4	*A. fumigatus*	28995825	49,47	461179	18	341	11956	98.9
MHA-5	*A. giganteus*	29202710	47,84	460550	17	150	12082	93.9
MHA-7*	*Flavi*	38510510	47,37	817189	16	507	14354	99.8
MHA-8	*Flavi*	38069066	47,37	618354	19	304	14027	100.0
MHA-9	*A. tubingensis*	35341942	49,07	732226	17	268	12687	99.8
MHA-10	*A. uvarum*	36155395	50,7	353053	30	387	15550	99.9
MHA-11	*Nigri*	37455823	49,35	630474	19	398	13107	99.6
MHA-12	*A. uvarum*	36233782	50,7	267144	47	474	15613	99.9
MHA-13	*A. fumigatus*	28338064	49,48	442112	22	213	11665	99.9
MHA-14	*Flavi*	37786859	47,12	988495	13	170	13805	99.9
MHA-15	*Flavi*	38155233	47,36	623322	19	410	14090	99.9
MHA-16*	*Flavi*	39703177	49,99	792746	17	843	14383	99.6
MHA-17	*A. amoenus*	34297266	49,93	1589780	7	301	15280	96.7
MHA-18	*Flavi*	37979300	47,31	604037	22	270	13948	99.9
MHA-19	*Nigri*	35824013	49,44	801508	16	222	12800	99.9
MHA-20	*A. tamarii*	38985472	47,14	529731	24	247	13743	99.9
MHA-21	*Nigri*	35543217	49,38	629228	16	289	12770	99.8

MHA-22	*A. ochraceus*	36668419	49,53	344704	32	390	15134	99.9
MHA-23	*A. sydowii*	35175103	49,87	1149476	10	421	15444	96.3
MHA-24	*Flavi*	38411394	47,22	635642	18	344	14173	99.8
MHA-25	*Nigri*	35891468	49,48	363817	29	288	12815	99.9
MHA-26	*A. tamarii*	38656385	47,25	455562	27	456	13869	99.9
MHA-27	*A. westerdijkiae*	36618508	48,93	335287	33	284	14948	97.3
MHA-30	*A. tubingensis*	35638552	48,74	1115806	12	116	12703	99.7
MHA-31*	*Nigri*	36953047	49,43	510221	24	502	13046	99.8
MHA-32*	*Nigri*	38066710	49,29	768660	17	830	13282	99.5
MHA-33	*A. tamarii*	38584510	47,27	481095	28	280	13754	99.9
MHA-35*	*Nigri*	35734723	49,48	723029	18	533	13915	93.7
MHA-36	*Nigri*	35859009	49,49	560462	23	309	12828	99.9
MHA-37	*A. tubingensis*	36319103	48,5	1048287	13	291	12766	99.7
MHA-38	*A. fumigatus*	28878622	49,45	419149	25	448	11952	99.7
MHA-39*	*Nigri*	37895509	49,34	822931	16	608	13179	99.9
MHA-40	*A. tamarii*	38788024	47,1	658982	19	461	13821	99.8
MHA-42	*Nigri*	35310406	49,55	378876	28	253	12719	99.9
MHA-43*	*A. fumigatus*	28982112	49,36	367257	23	922	12047	99.6
MHA-44	*A. tamarii*	38398742	47,12	519754	24	208	13613	99.9
MHA-45	*A. tubingensis*	35381244	49,07	731063	16	258	12647	99.9
MHA-46	*Flavi*	38061355	47,37	403104	30	352	14018	100.0
MHA-47*	*Flavi*	38605236	47,33	725553	18	1951	14802	99.5
MHA-49	*A. terreus*	30397284	52,09	1065513	9	448	14905	99.9
MHA-50*	*A. tubingensis*	37091698	49,02	614963	21	3026	13275	99.8
MHA-51	*A. terreus*	30310758	52,14	1184609	10	301	14931	99.7
MHA-52	*A. fumigatus*	28536676	49,45	406002	25	327	11785	99.8
MHA-53	*A. fumigatus*	29133377	49,4	320319	26	377	11767	99.9
MHA-54	*A. fumigatus*	28564715	49,45	408991	25	304	11704	99.9
MHA-55	*Nigri*	36559342	49,47	674164	17	328	12967	99.9
MHA-56	*A. fumigatus*	29057505	49,38	331313	28	368	11768	99.9
MHA-57	*A. fumigatus*	28226360	49,48	340474	24	273	11618	99.8
MHA-58	*A. fumigatus*	28231447	49,47	327180	25	261	11625	99.9
MHA-59	*A. fumigatus*	28240885	49,47	327819	25	279	11624	99.8
MHA-61	*Flavi*	37868225	47,3	659021	19	211	13964	100.0
MHA-62	*Flavi*	37155050	47,7	577007	21	477	13879	99.9
MHA-64	*Nigri*	35823408	49,48	439378	25	256	12771	99.9
MHA-65	*A. tubingensis*	35491348	49,46	550207	21	477	12850	99.8
MHA-66	*A. fumigatus*	28218469	49,48	326539	28	260	11666	99.5
MHA-67	*A. fumigatus*	28635696	49,51	435538	24	240	11735	99.9
MHA-69	*A. fumigatus*	28665041	49,42	333294	28	218	11717	99.8
MHA-71	*A. fumigatus*	28837893	49,5	419666	23	279	11813	99.8
MHA-73	*A. fumigatus*	28818161	49,51	419382	22	260	11782	100.0
MHA-77	*A. fumigatus*	28300579	49,46	374625	26	212	11628	99.9
MHA-78	*A. fumigatus*	28114869	49,52	411607	22	189	11588	99.8

MHA-79	*A. fumigatus*	28105964	49,52	405431	22	200	11586	99.9
MHA-80	*A. fumigatus*	28836676	49,5	427559	21	269	11791	99.9
MHA-81	*A. fumigatus*	28048564	49,54	540263	18	169	11591	99.8
