# Supplementary material for: Species Identification and Orthologous Allergen Prediction and Expression in the Genus Aspergillus
Source: J Fungi (Basel). 2025 Jan 27;11(2):98. doi: 10.3390/jof11020098 (PMC11856533; doi:10.3390/jof11020098)
Supplement: Supplementary file 1 [file jof-11-00098-s001.zip › Table S7.pdf]

Table S7. Allergens officially recognized to *Aspergillus* species by WHO/IUIS Allergen Nomenclature Sub-Committee.

Section	Allergen	Biochemical name	GenBank gene	pb	kDa	# aa	GenBank Protein
Fumigati	Asp f 1	Mitogillin/ribonoclease toxin	Afu5g02330	583	19,6	176	XP_748109.1
	Asp f 2	Allergen Asp f 2	Afu4g09580	998	32,8	304	XP_751868.1
	Asp f 3	Peroxiredoxin	Afu6g02280	747	18,4	168	XP_747849.1
	Asp f 4	Allergen Asp f 4	Afu2g03830	969	30,0-34,0	322	XP_749515.1
	Asp f 5	Metalloprotease	Afu8g07080	2111	68,7	634	XP_747506.1
	Asp f 6	Mn superoxide dismutase	Afu1g14550	748	23,4	210	XP_752824.1
	Asp f 7	Alérgeno Allergen Asp f 7	Afu4g06670	813	11,6-27,5	270	XP_752159.1
	Asp f 8	60S Acidic ribosomal protein P2	Afu2g10100	407	11,1	111	XP_755343.1
	Asp f 9	Extracellular cell wall glucanase Crf1	AFUA_1G16 190	1303	32,3-40,2	395	XP_752985.1
	Asp f 10	Aspergillopepsin F/ aspartic protein	Afu5g13300	1348	41,6	395	XP_753324.1
	Asp f 11	Peptidyl-prolyl cis-trans isomerase/cyclophilin	Afu2g03720	875	19,5	205	XP_749504.1
	Asp f 12	Molecular chaperone Hsp90	Afu5g04170	2182	50,5-80,3	706	XP_747926.1
	Asp f 13	Allergenic cerato-platanin	Afu2g12630	459	15,9	152	XP_755595.1
	Asp f 15	Cerato-platanin (Simitar to Asp f 13)					
	Asp f 16	Glucosidase (Similar to Asp f 9)					
	Asp f 17	Cell wall serine-threonine-rich galactomannoprotein Mp1	Afu4g03240	855	19,4-27,3	284	XP_746510.1
	Asp f 18	Serine protease/ autophagic serine protease Alp2	Afu5g09210	1623	52,6	495	XP_753718.1
	Asp f 22	Enolase	Afu6g06770	1501	47,3	439	XP_750570.1
	Asp f 23	60 S ribosomal L3 protein	Afu2g11850	1540	44,4	392	XP_755517.1
	Asp f 27	Putative peptidyl-prolyl cis-trans isomerase	Afu3g07430	704	18	163	XP_754866.1
	Asp f 28	Tiorredoxin, putative	Afu6g10300	379	13	108	XP_750918.1

	Asp f 29	Tiorredoxin TrxA	Afu5g11320	412	13	110	XP_753517.1
	Asp f 34	Cell wall protein PhiA	Afu3g03060	622	20	185	XP_748628.1
	Asp f 35	Cu-Zn Superoxide dismutase similar to Ole e 5	MZ731825.1	165	21	154	UYL70859.1
	Asp f 36	Fructose-bisphosphate aldolase	AFUA_3G11690	1278	42	360	XP_754452.1
	Asp f 37	Malate dehydrogenase NAD-Dependent	AFUA_6G05210	1441	35	330	XP_747556.1
	Asp f 38	Uncharacterized Protein	AFUA_5G14680	684	25	227	XP_753189.1
	Asp f 39	FG-GAP repeat protein	AFUA_1G04130	924	35	307	XP_750162.2
Flavi	Asp o 13	Orizin / Alkaline serine protease	AO090003001036	1377	34	403	XP_001820144.1
	Asp o 21	α-amilase A	AO090023000944	2935	54	499	XP_001821436.1
	Asp fl 13	Alkaline serine protease	G4B84_003708	1377	34	403	XP_041143422.1
Nigri	Asp n 14	β-xilosidase	An01g09960	2548	105	804	XP_001389416.1
	Asp n 18	Vacuolar serine protease	ANI_1_476064	2065	34	497	XP_001391470.1
	Asp n 25	3 – Fitase B	An08g11030	1836	66-100	479	XP_001393206.1
Nidulantes	Asp v 13	Extracelullar Alkaline Serine Protease	ASPVEDRAFT_29272	1382	43	403	XP_040668476.1
Terrei	Asp t 36	Triosephosphate Isomerase	ATEG_06163	1122	27	249	XP_001215341.1
