# Supplementary figures and images for: Species Identification and Orthologous Allergen Prediction and Expression in the Genus Aspergillus
Source: J Fungi (Basel). 2025 Jan 27;11(2):98. doi: 10.3390/jof11020098 (PMC11856533; doi:10.3390/jof11020098)

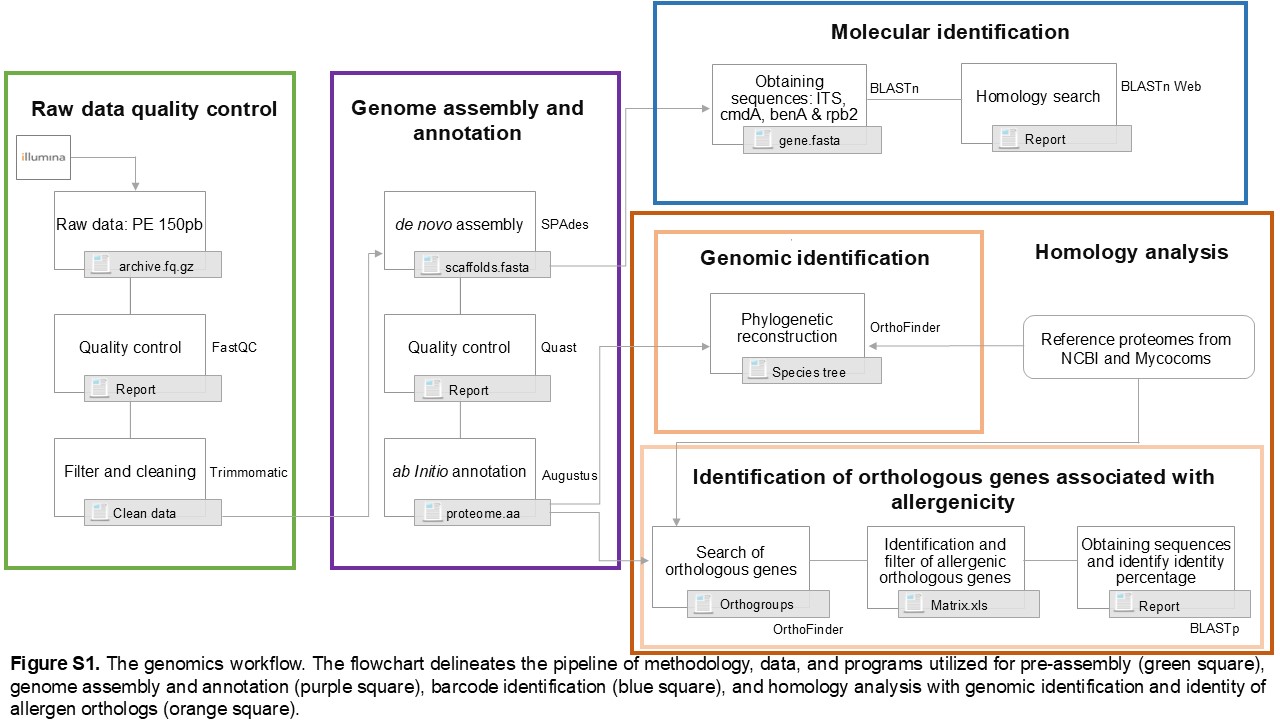

Supplement: Supplementary file 1 [file jof-11-00098-s001.zip › Figure S1.jpg]

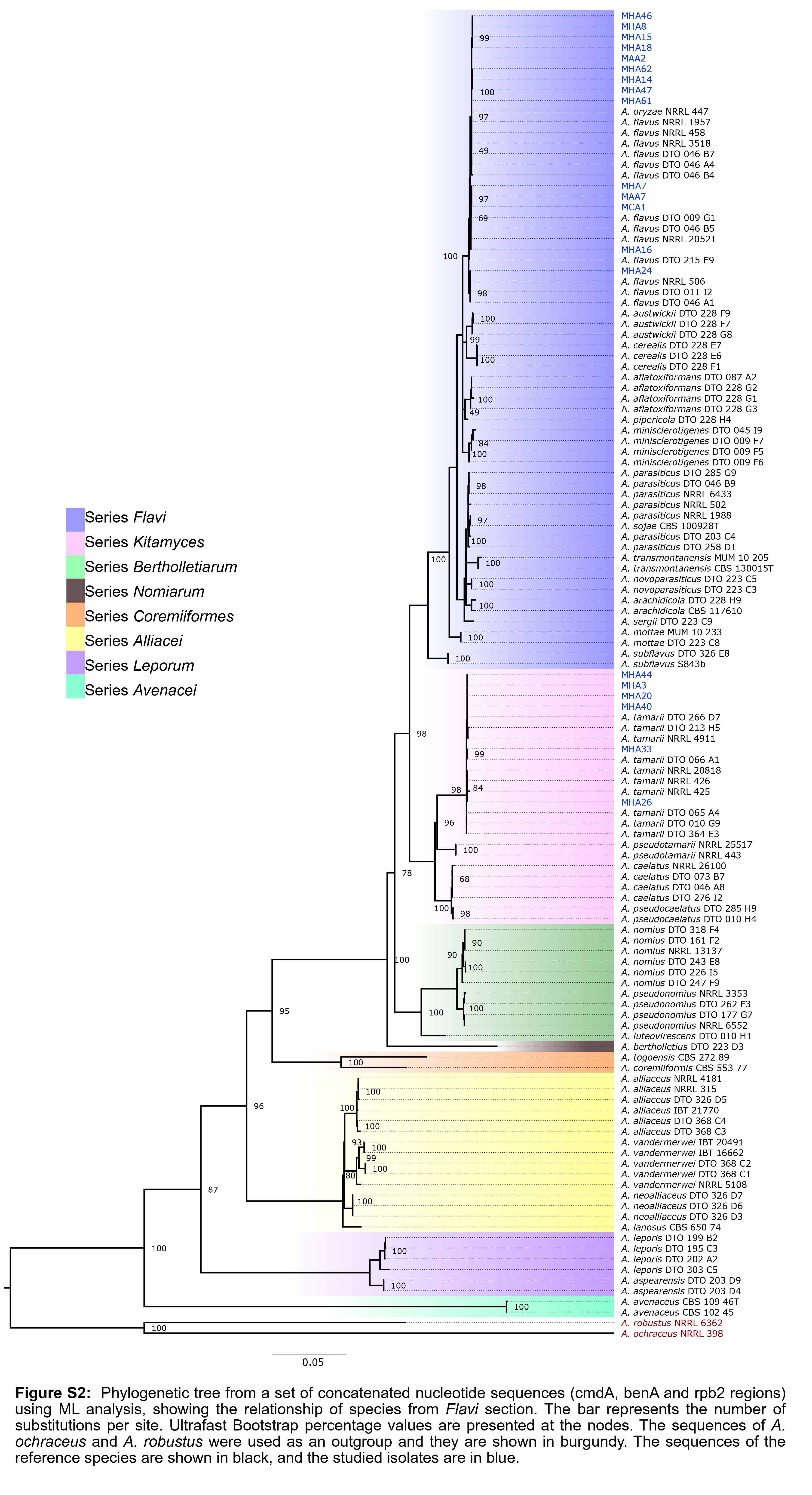

Supplement: Supplementary file 1 [file jof-11-00098-s001.zip › Figure S2.jpg]

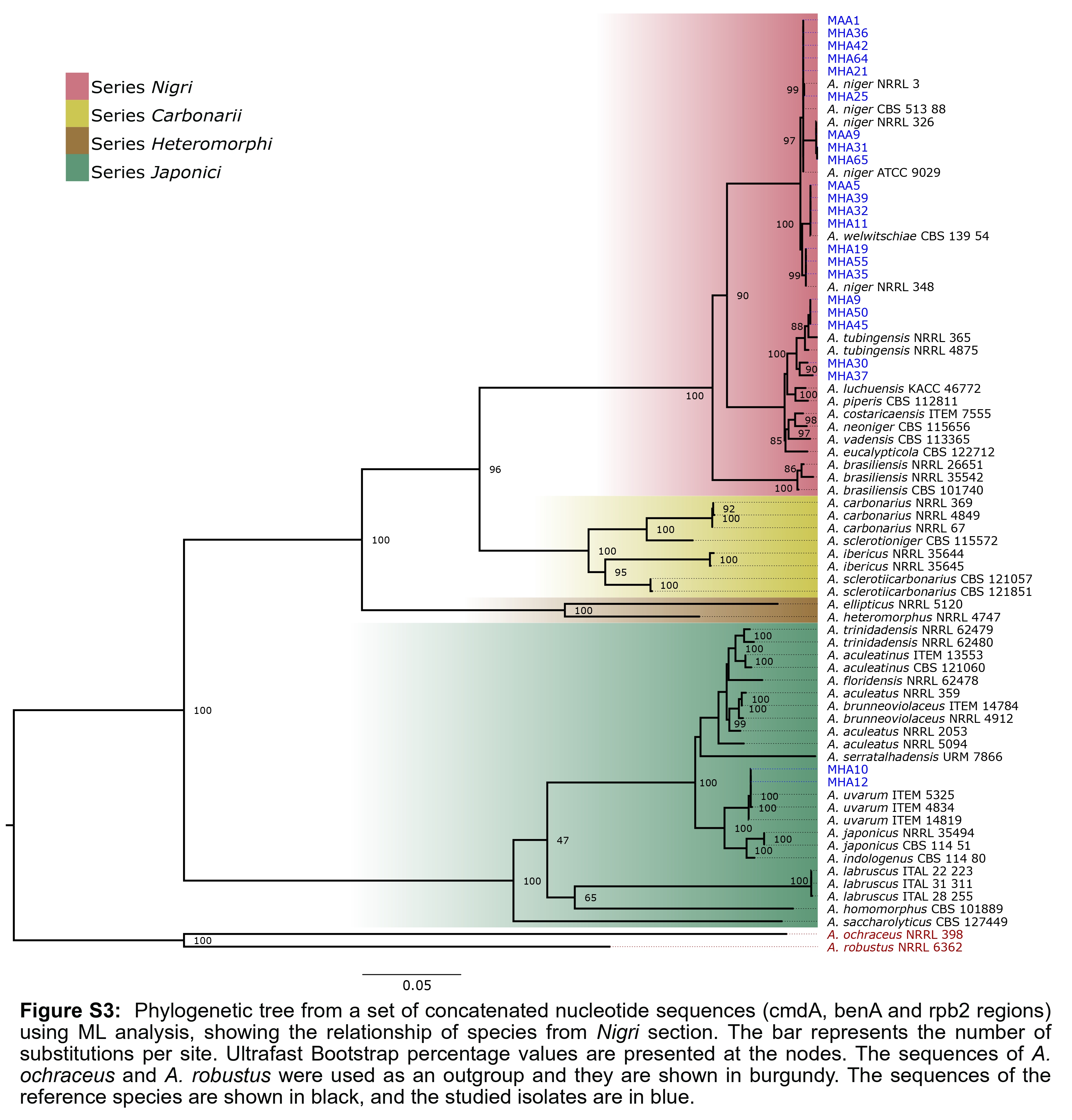

Supplement: Supplementary file 1 [file jof-11-00098-s001.zip › Figure S3.jpg]

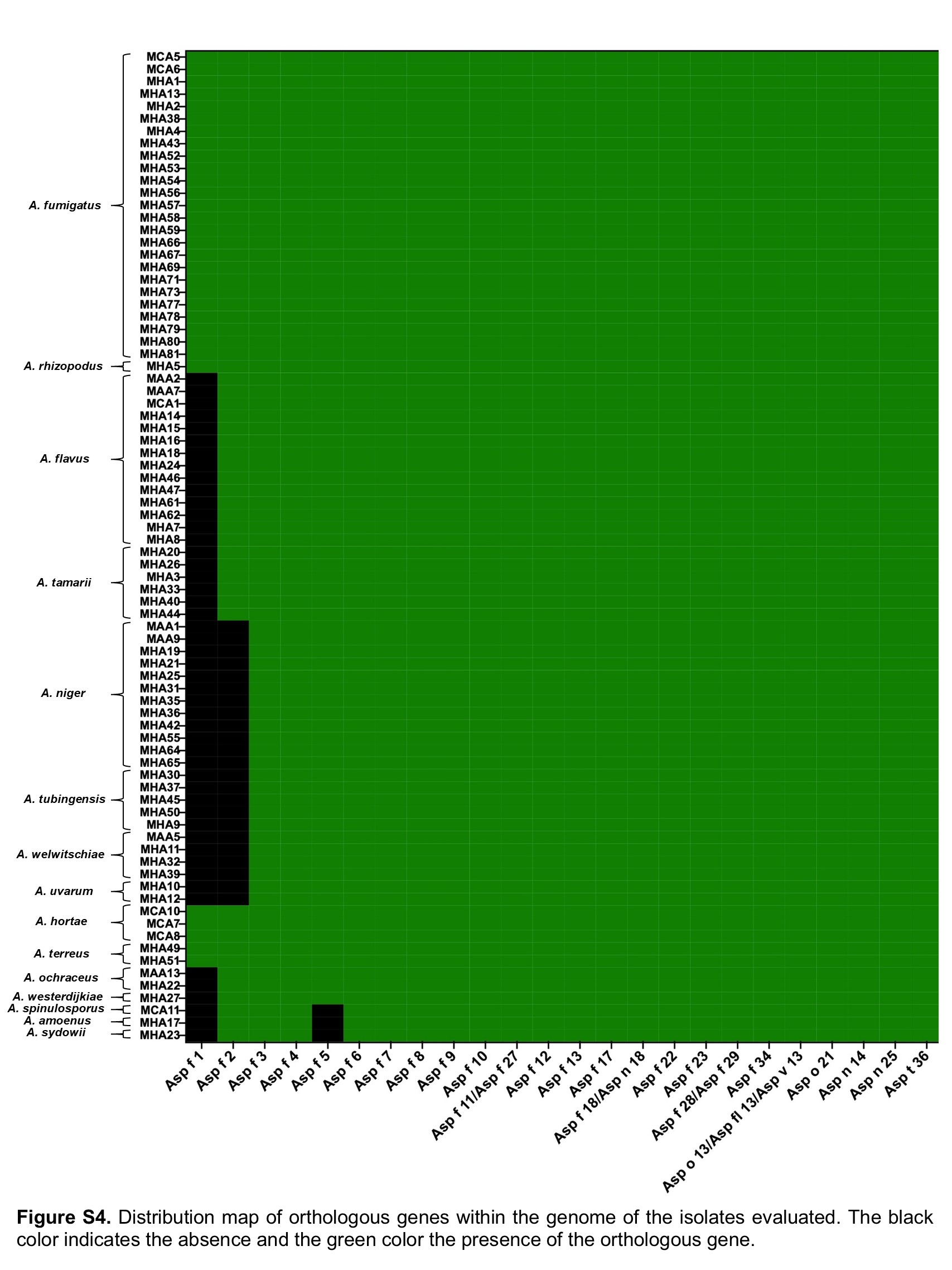

Supplement: Supplementary file 1 [file jof-11-00098-s001.zip › Figure S4.jpg]

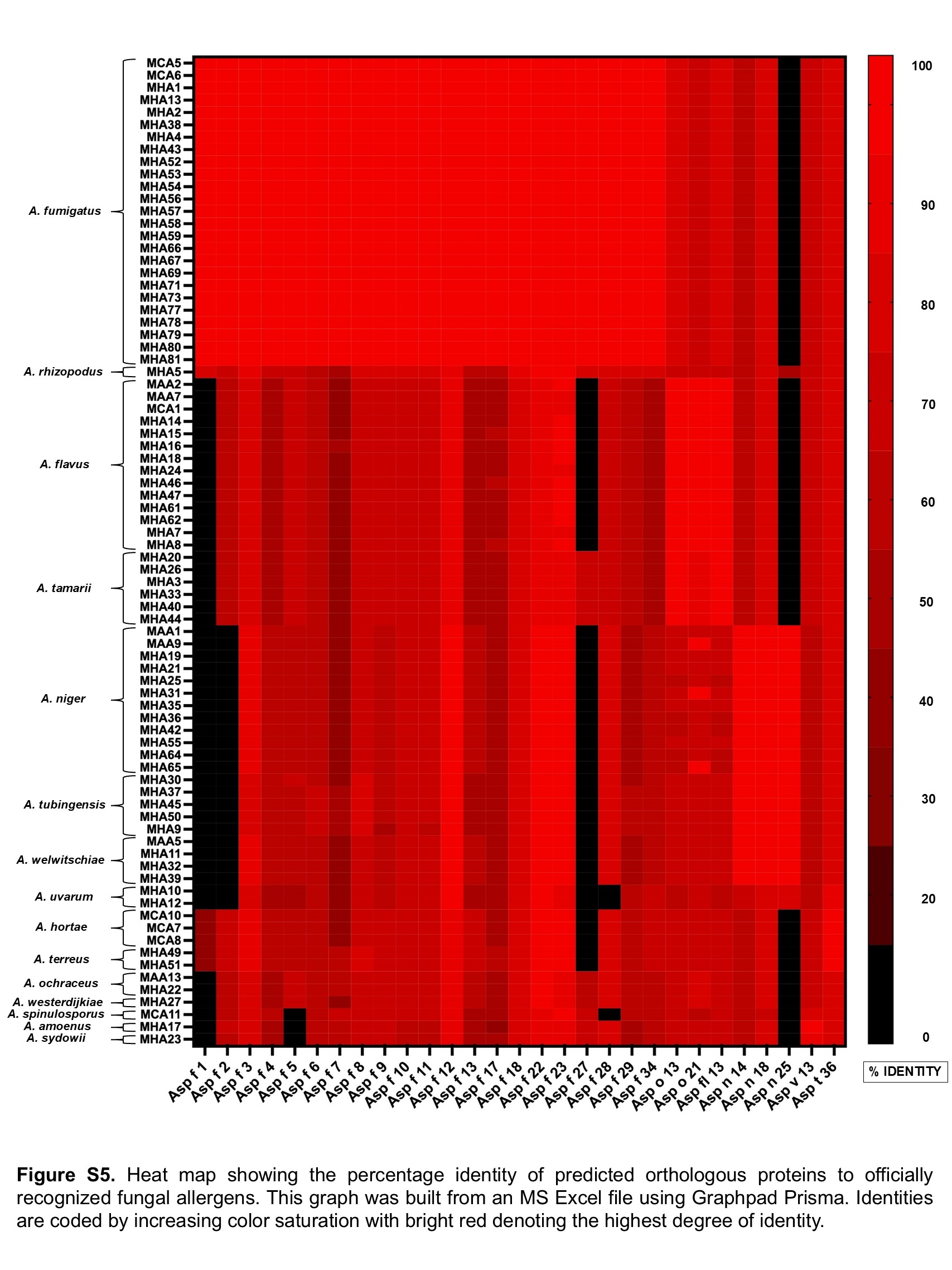

Supplement: Supplementary file 1 [file jof-11-00098-s001.zip › Figure S5.jpg]

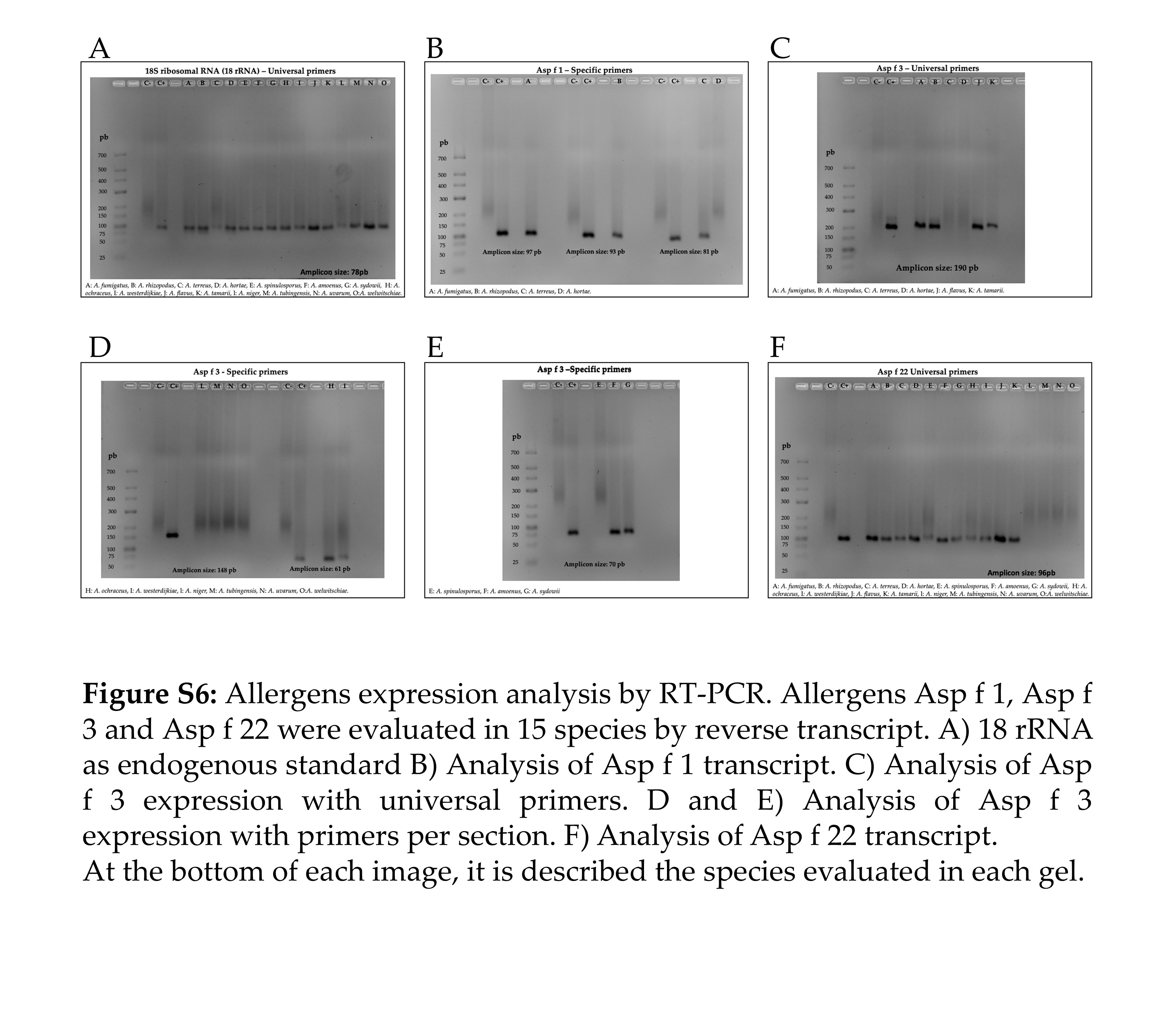

Supplement: Supplementary file 1 [file jof-11-00098-s001.zip › Figure S6.jpg]
